# Comparison between SBMA and MPC coatings on PEEK surface: stability over time and anti-inflammatory effects in vitr﻿o

**DOI:** 10.1038/s41598-025-25082-5

**Published:** 2025-11-21

**Authors:** Erika Roventini, Francesco Iacoponi, Aliria Poliziani, Paolo Canepa, Ornella Cavalleri, Maria A. Cassa, Paola Parlanti, Carlotta Pucci, Mauro Gemmi, Chiara Tonda-Turo, Leonardo Ricotti

**Affiliations:** 1 The BioRobotics Institute, Scuola Superiore Sant’Anna, Piazza Martiri della Libertà, 33, 56127 Pisa, Italy; 2 Department of Excellence in Robotics & AI, Scuola Superiore Sant’Anna, Piazza Martiri della Libertà, 33, 56127 Pisa, Italy; 3 Department of Physics, University of Genoa, Dodecaneso, 33, 16146 Genoa, Italy; 4 Department of Mechanical and Aerospace Engineering, Politecnico Di Torino, Corso Duca degli Abruzzi, 24, 10129 Turin, Italy; 5 PolitoBIOMed Lab, Politecnico Di Torino, Corso Castelfidardo, 30A, 10129 Turin, Italy; 6 Istituto Italiano di Tecnologia, Center for Materials Interfaces, Electron Crystallography, Viale Rinaldo Piaggio, 34, 56025 Pontedera, Italy

**Keywords:** 2-methacryloyloxyethyl phosphorylcholine, [2-(methacryloyloxy)ethyl]dimethyl-(3-sulfopropyl)ammonium hydroxide, Implantable device, Macrophage, Chemistry, Materials science, Medical research

## Abstract

The foreign body reaction often hinders the success of implantable devices, leading to fibrotic encapsulation. Controlling the post-implantation inflammatory phase is key to mitigating the foreign body reaction and promoting device integration. This paper compares three coating strategies for including anti-inflammatory agents onto polyether-ether-ketone (PEEK) surfaces, applying two different zwitterions, namely the 2-methacryloyloxyethyl phosphorylcholine (MPC) and [2-(methacryloyloxy)ethyl]dimethyl-(3-sulfopropyl)ammonium hydroxide (SBMA). Two different PEEK surface types were also compared, namely “rough surface” and “smooth surface” (average roughness: 7 μm ± 4 μm and 1.4 μm ± 0.3 μm, respectively). Focused ion beam-scanning electron microscopy (FIB-SEM) confirmed qualitatively the presence of a thin coating layer with an estimated thickness of 100 nm. X-ray photoelectron spectroscopy and water contact angle measurements showed that both MPC and SBMA coatings were highly stable on the PEEK substrates for up to 8 weeks in simulated physiological conditions, when anchored to the PEEK surfaces by exploiting zwitterionic copolymers with N-[3-(dimethylamino)propyl]acrylamide and using a polydopamine adhesive layer. These coatings were also tested in vitro, evaluating their effects on cell adhesion, and the production of inflammatory markers such as interleukin-1β, interleukin-6, tumor necrosis factor-α and nitric oxide from M1 macrophages. The MPC-based coating on smooth PEEK surfaces showed the most remarkable effects, significantly supporting macrophage viability, reducing the release of pro-inflammatory cytokines, and inhibiting nitric oxide release, suggesting a superior capability to modulate inflammation. These findings pave the way for functional coatings to be used in vivo, with the aim of improving PEEK implants’ safety, integration, and longevity.

**Supplementary Information:**

The online version contains supplementary material available at 10.1038/s41598-025-25082-5.

## Introduction

In recent years, the demand for implantable devices has steadily increased to tackle different pathological conditions, including cardiac dysfunctions^1^, diabetes^2^, neural disorders^3^, and respiratory deficiencies^4^, among many others.

However, when a medical device is implanted, it is subjected to a series of events known as the foreign body reaction (FBR). The FBR starts with the non-specific adsorption of proteins onto the surface. This is followed by an acute inflammatory phase, characterized by the infiltration of immune cells, primarily monocytes, which differentiate into macrophages on the device surface. During the early phase of inflammation, these macrophages (M0) adopt a pro-inflammatory phenotype (M1) to combat the foreign body and external pathogens. Macrophages then release cytokines and chemokines, such as tumor necrosis factor-α (TNF-α), interleukin-6 (IL-6), and interleukin-1β (IL-1β)^5^, which attract fibroblasts to the site, leading to the formation of a fibrous capsule all around the device. At this stage, if the subsequent chronic state of inflammation is resolved, the device is integrated into the body. Conversely, if chronic inflammation persists for more than 3 weeks^5,6^ the implantation may fail due to an excessive fibrotic response that can lead to the replacement of the implanted device, often resulting in complications for patients and high costs for healthcare systems.

The oral administration of anti-inflammatory molecules is widely used to control inflammation. Conventional drugs (such as steroidal and non-steroidal drugs, antibodies, and biological agents) or natural compounds (such as polyphenols, flavonoids, and triterpenes) are typically administered after a surgical procedure^7,8^. However, their administration at the systemic level may not achieve the desired local concentration at the target site, and their long-term use may induce side effects.

Surface modification of the implant by using macromolecules represents a valid alternative. Among these, the most commonly used are glycosaminoglycans (heparin and hyaluronic acid); however, their biological origin may lead to undesired immune reactions^9,10^. Polyethylene glycol (PEG) is also often used; however, it can be subjected to rapid autoxidation in a physiological environment, tends to bioaccumulate in cell lysosomes, and can lead to the production of anti-PEG antibodies^11^. Polymers based on zwitterionic molecules have been considered to overcome the limitations of PEG^12^. Zwitterions are electrically neutral molecules, with an arrangement that mimics biological structures, such as membrane phospholipids^13^, or the amino acid sequence arginine-glycine-aspartic acid, which contains zwitterionic units under physiological conditions^14^. In contrast to PEG, zwitterions have high salt tolerance^14^ and stability, as well as ultra-low immunogenicity and antigenicity^15^. However, their application to PEEK surfaces has only been preliminarily explored so far.

PEEK is a semi-crystalline thermoplastic polymer that attracts increasing attention in the biomedical field due to its high thermal stability, high resistance to oxidation, excellent mechanical properties, fatigue resistance, and good biocompatibility^16^. These are just a few features that make PEEK ideal for use in implantable devices^17–19^. In particular, thanks to its elastic modulus (3–4 GPa), which is comparable to that of bone tissue (human cortical bone: 7–30 GPa)^18,20^, it is widely used in medical fields, such as orthopedics, maxillofacial surgery, and dentistry^21,22^. Furthermore, due to its printability, highly customized shapes and geometries can be achieved using fused deposition modeling (FDM) techniques^23^. Thanks to its flexibility, it can be used to fabricate heart valve prostheses and leaflet heart valves^24^. Despite these advantages, the major limitation of PEEK is its biological inertness, which leads to poor integration with surrounding tissues and can provoke an acute inflammatory response upon implantation^25^. When not properly regulated, this response may promote fibrosis and encapsulation, compromising device functionality and long-term performance^2^. Therefore, surface functionalization becomes essential. In addition, the chemical stability of PEEK makes it particularly challenging to modify, as it resists most chemical reactions required for introducing functional groups onto its surface^26^. The fabrication of an adhesive intermediate layer that can be further functionalized with zwitterionic molecules can overcome this challenge^27,28^. For this purpose, a biomimetic approach, inspired by the adhesive versatility shown by marine mussels, has been used. The amino acid composition of the adhesive plaque of marine mussels is responsible for this peculiarity, particularly due to the presence of 3,4-dihydroxy-L-phenylalanine and lysine, with their functional groups, catechol and amine groups, respectively^29^. To reproduce the effect of marine mussels, dopamine, which contains the same functional groups, was selected to fabricate a strong adhesive primer on the PEEK substrate via an efficient dip-coating technique that can be further functionalized with zwitterionic molecules^29^. Recent studies have further highlighted the potential of PDA functionalization on PEEK to improve osteogenic and antibacterial properties, confirming its versatility as a bioactive platform^30,31^.

Surface roughness is a crucial factor influencing the biological performance of implantable materials. Microscale and nanoscale topographical features can modulate cell adhesion and inflammatory responses, often enhancing osteointegration or soft tissue attachment depending on the application^32,33^. However, increased roughness may also promote macrophage activation and fibrotic responses, especially in the absence of surface bioactivity^34^. For this reason, comparing rough and smooth PEEK surfaces is essential to assess how zwitterionic coatings perform under different topographical conditions that mimic realistic implant environments.

Despite the above-mentioned exciting results, a systematic comparison between different zwitterions applied to PEEK surfaces, evaluating their stability over time and their anti-inflammatory properties, is currently missing from the state-of-the-art. In this study, two different zwitterions, SBMA and MPC, were used to modify PEEK substrates through three different chemical strategies. Among these, the first two approaches were adapted from the literature. In contrast, the third one represents an innovative strategy introducing a co-monomer that stabilizes the zwitterionic chains, accentuates and preserves the hydrophilic character of the coating under physiological conditions^35^. As noted in the literature, hydrophilic substrates can modulate macrophage polarization^36–38^. For instance, Hotchkiss et al. reported that the expression of the principal pro-inflammatory cytokine genes (e.g. IL-1β, IL-6, TNF-α) was reduced in the hydrophilic Titanium-zirconium substrate compared with the hydrophobic counterpart^39^.

Moreover, we performed a systematic comparison considering two different surface roughness levels of the PEEK surfaces, to investigate the role of the substrate topography in influencing coating performance. The coatings were compared in terms of stability over time under physiological conditions, macrophage and fibroblast adhesion, and ability to reduce the production of inflammatory markers by M1 macrophages.

## Results and discussion

### Characterization of PEEK surfaces

Disc-shaped PEEK samples were produced using the FDM technique. Two different surface finishes were considered, leading to different roughness levels (referred to as ROUGH and SMOOTH samples, respectively). In 3D-printed PEEK, surface topography can vary depending on the post-processing applied, with as-fabricated substrates generally displaying higher roughness and post-processed substrates being significantly smoother. Since roughness has been reported to influence macrophage activation and fibrotic reactions even when the differences are within the micrometer scale^40–42^, comparing ROUGH and SMOOTH PEEK surfaces is essential to evaluate whether zwitterionic coatings maintain their stability and immunomodulatory effects under distinct and clinically relevant topographical conditions. However, no information is available concerning the effect of roughness on the long-term stability (up to 8 weeks) and anti-inflammatory efficacy of zwitterionic coatings. The inclusion of substrates with varying roughness values in our analyses allowed us to gain insights into these aspects.

**Fig. 1:**
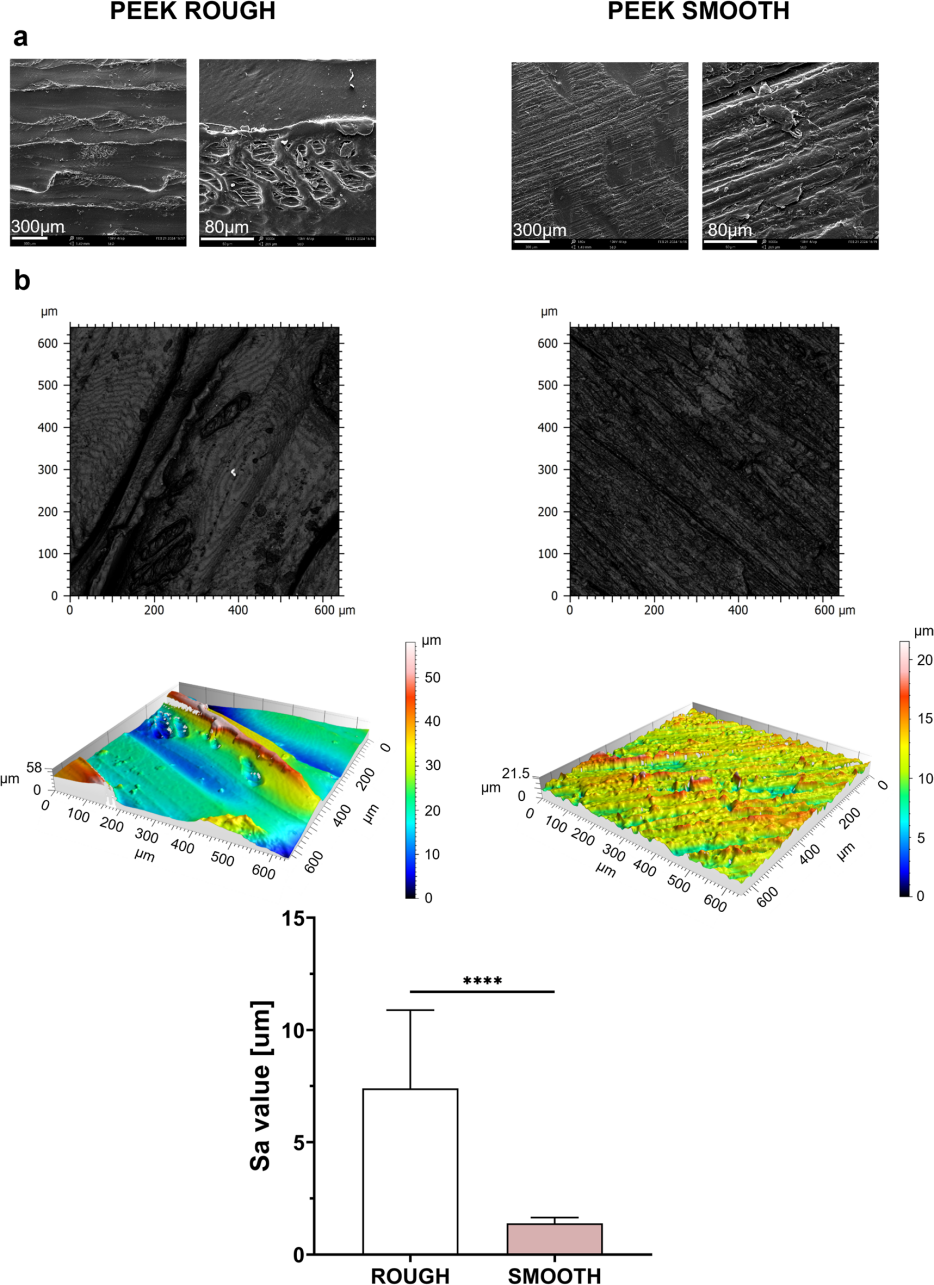
Surface characterization: (**a**) SEM images of PEEK ROUGH and PEEK SMOOTH surfaces. (**b**) Profilometer 2D and 3D maps for PEEK ROUGH and PEEK SMOOTH surfaces, and a graph reporting the comparison between their Sa. **** = *p* < 0.0001. PEEK = polyether-ether-ketone, Sa = average surface roughness.

The hydrophobicity of PEEK was assessed by water contact angle (WCA) measurements. The WCA values for PEEK ROUGH (85° ± 4°, mean ± std) and PEEK SMOOTH (92° ± 8°) were not statistically different and were comparable to those reported in the literature, confirming the typical hydrophobic behavior of PEEK substrates^20,21^. Scanning electron microscopy (SEM) images (Fig. 1a) revealed different morphologies. On the ROUGH samples, characteristic grooves produced by the FDM technique used to fabricate the PEEK discs were clearly visible. On the PEEK SMOOTH samples, which underwent post-processing with abrasive paper, a still-irregular but more flattened surface was observed. The results of profilometry characterization are shown in Fig. 1b. The images confirmed the qualitative considerations derived from SEM images. Furthermore, quantitative results highlighted that the average surface roughness (Sa) of PEEK ROUGH samples (Sa: 7 μm ± 3 μm, mean ± std) was significantly higher (*p* < 0.0001) than that of PEEK SMOOTH samples (Sa: 1.4 μm ± 0.3 μm).

### Characterization of zwitterionic coatings and their stability over time

**Fig. 2:**
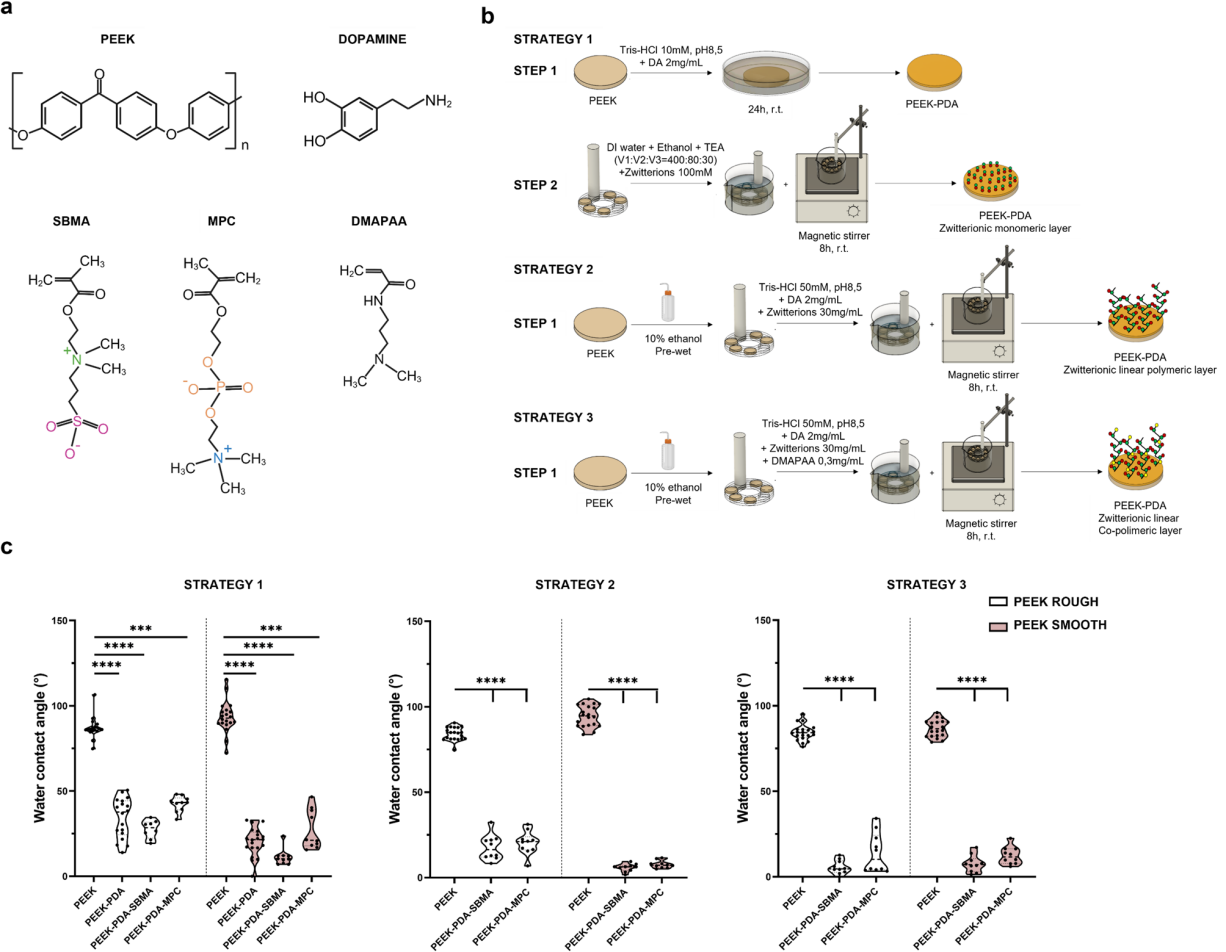
Depiction and characterization of zwitterionic coatings on PEEK substrates. (**a**) Chemical structures of the molecules involved. (**b**) Schematic description of the three chemical strategies used to functionalize the PEEK surfaces with the two zwitterions. (**c**) WCA measurements on bare PEEK samples and the coated ones. *** = *p* < 0.001, **** = *p* < 0.0001. PEEK = polyether-ether-ketone, SBMA = [2-(methacryloyloxy)ethyl]dimethyl-(3-sulfopropyl)ammonium hydroxide, MPC = 2-methacryloyloxyethyl phosphorylcholine, PDA = polydopamine, DMAPAA = N-[3-(dimethylamino)propyl]acrylamide].

Two zwitterions were used to modify the surface of PEEK substrates, namely SBMA and MPC. For each of them, three different chemical strategies were used to functionalize the PEEK surface (Fig. 2a, b).

The increased hydrophilicity of the PEEK surface after coating served as indirect evidence confirming the presence of zwitterions, as highlighted by Kyomoto et al.^43^. In our case, a significant decrease in WCA values was observed after adding the SBMA and MPC coatings using the three strategies tested (Fig. 2c).

As shown in Fig. 2c, after the first step of strategy 1, namely the addition of a polydopamine (PDA) layer, a significant decrease in the WCA values was found (PEEK ROUGH changed from 86°(85–88) to 38°(24–44), PEEK SMOOTH changed from 93°(90–97) to 22°(17–26); values expressed as median(interquartile range, IQR)). These findings are consistent with those available in the literature, where the same PDA-based coating strategy was used to functionalize titanium substrates^44,45^. The observed WCA reduction can be attributed to the presence of hydrophilic functional groups (e.g., catechol and amine groups) introduced by the PDA layer, which enhance surface wettability and promote better interaction with aqueous environments^46^. The hydrophilicity achieved was maintained after the second step of functionalization. For PEEK ROUGH, the coating based on SBMA led to a WCA of 29°(22–32), while the coating based on MPC led to a WCA of 43°(39–46). For PEEK SMOOTH, the coating based on SBMA produced a WCA of 10°(8–12), and the coating based on MPC produced a WCA of 21°(18–39).

For the samples functionalized with strategy 2 and strategy 3 (Fig. 2c), the significant change (*p* < 0.0001) in WCA values after adding the coatings (based on SBMA and MPC) confirmed the presence of a hydrophilic layer formed by zwitterions and PDA. Notably, we observed that when strategy 3 was applied, WCA values showed lower median values for PEEK ROUGH modified with SBMA and MPC compared to the other strategies. For PEEK SMOOTH, the median values obtained with strategy 3 were lower than those obtained with strategy 1 but slightly higher than those obtained with strategy 2.

X-ray photoelectron spectroscopy (XPS) analyses were conducted to study the composition of the coatings and to demonstrate the presence of zwitterions^47^. The data reported in Supplementary Fig. S1 and Supplementary Fig. S2 show the typical XPS signals of the molecules used, namely PDA, N-[3-(dimethylamino)propyl]acrylamide] (DMAPAA), SBMA, and MPC, as well as those of the coatings on PEEK samples after their functionalization with strategies 1, 2, and 3. These data confirm the presence of the expected molecules in all the coatings.

**Fig. 3:**
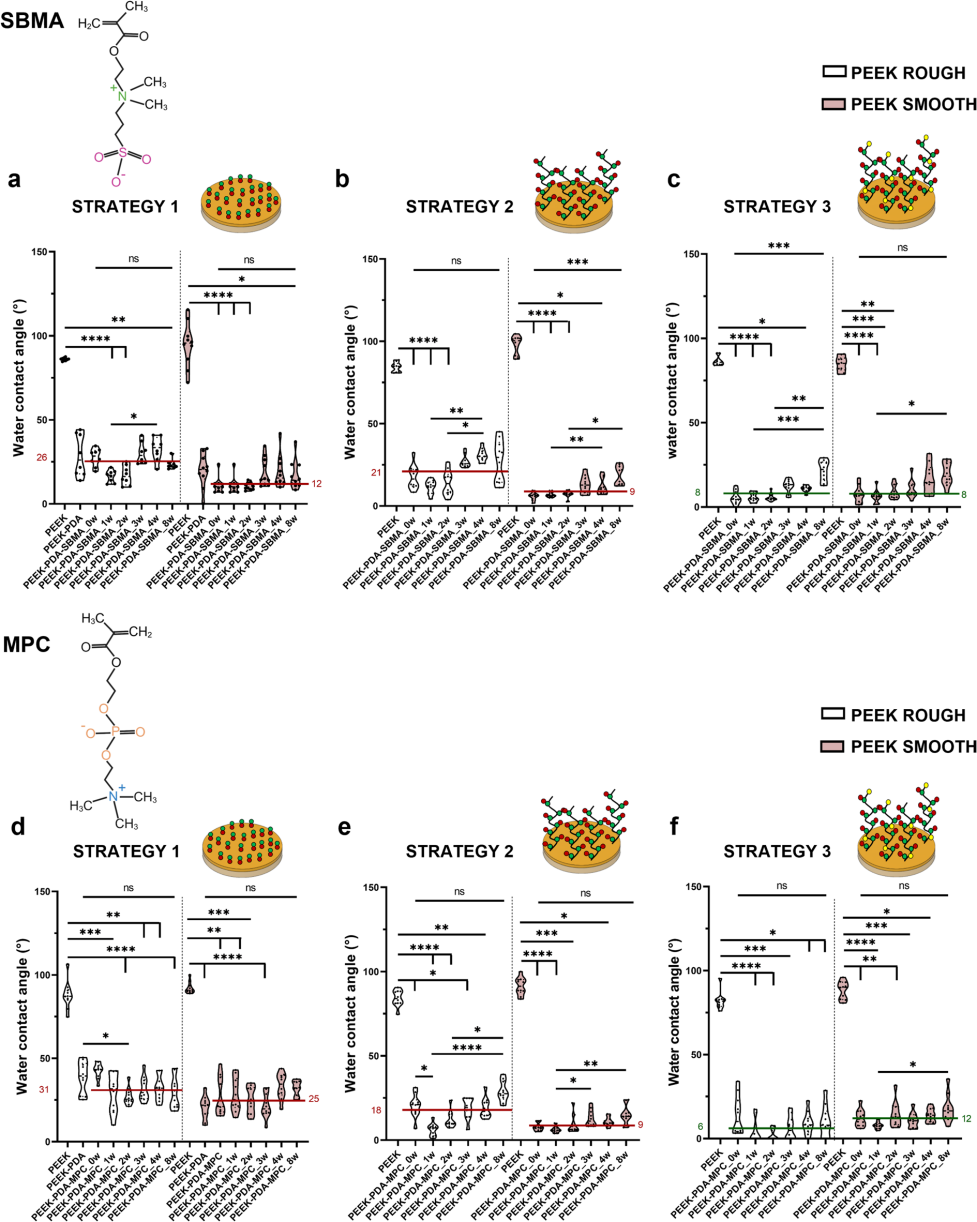
Coating characterization and stability over time. Evaluation of the WCA measured before and after the coating deposition, and at different time points (1 week, 2 weeks, 3 weeks, 4 weeks, and 8 weeks) following storage under physiological conditions (samples immersed in PBS at 37 °C, 200 rpm oscillation). SBMA-based coating fabricated with (**a**) Strategy 1, (**b**) Strategy 2, (**c**) Strategy 3 and MPC-based coating fabricated with (**d**) Strategy 1, (**e**) Strategy 2, (**f**) Strategy 3. * = *p* < 0.05, ** = *p* < 0.01, *** = *p* < 0.001, **** = *p* < 0.0001. PEEK = polyether-ether-ketone, SBMA *=* [2-(methacryloyloxy)ethyl]dimethyl-(3-sulfopropyl)ammonium hydroxide, MPC = 2-methacryloyloxyethyl phosphorylcholine, PDA = polydopamine.

WCA measurements were also used to evaluate the coating stability under physiological conditions. Samples were kept in 1X phosphate-buffered saline (PBS) at 37 °C under continuous shaking at 200 rpm for up to 8 weeks, providing information that was previously unavailable in the literature. As shown in Fig. 3a, the SBMA-based coating obtained through strategy 1 on PEEK ROUGH and PEEK SMOOTH remained stable after 8 weeks: the difference between the PEEK samples coated and tested immediately after coating application (labeled in the figure as PEEK-PDA-SBMA_0w) and those coated and kept for 8 weeks under physiological conditions (in the figure labeled as PEEK-PDA-SBMA_8w) was not significant for any of the three strategies adopted. Interestingly, the median(IQR) of WCA values (26°(17–30) for PEEK ROUGH and 12°(10–15) for PEEK SMOOTH) was slightly higher than that typically found in the literature for zwitterionic coatings, which are below 10°^15,43,48^.

With strategy 2 (Fig. 3b), PEEK ROUGH showed WCA values larger than those typically found in the literature, while PEEK SMOOTH showed WCA values with a lower median value (9°(6–14)). Strategy 3 (Fig. 3c) led to WCA values with a median < 10° for both PEEK ROUGH (8°(5–16)) and PEEK SMOOTH (8°(7–15)).

As shown in Fig. 3d–f, the coating based on MPC obtained through all three strategies remained stable after 8 weeks, both for PEEK ROUGH and PEEK SMOOTH substrates (for all samples, PEEK-PDA-MPC_0w and PEEK-PDA-SBMA_8w showed no statistically significant differences).

For PEEK ROUGH, strategy 3 showed less variability and a median value smaller than the other strategies (WCA median(IQR) in strategy 1: 31°(27–35), in strategy 2: 18°(9–23), in strategy 3: 6°(0–9)). For PEEK SMOOTH, strategy 2 and strategy 3 showed a smaller median value compared to strategy 1 (WCA median(IQR) in strategy 1: 25°(21–33), in strategy 2: 9° (6–12), in strategy 3: 12°(10–15)).

Strategy 1 and strategy 2 have already been described in the literature to functionalize substrates with zwitterionic coatings^15,44,45,47^. However, in those cases, coating stability was not assessed for up to 8 weeks. Strategy 3, on the other hand, was never proposed before for implementing zwitterionic coatings. Such a strategy relies on the deposition of dopamine and a co-polymer made of the zwitterionic and DMAPAA monomers, using a solution enriched with DMAPAA. Interestingly, considering the WCA median values of all the sample types (PEEK ROUGH and PEEK SMOOTH, for both SBMA and MPC coatings), strategy 3 was the one that guaranteed, on average, the greatest hydrophilicity (the overall median value for strategy 3: 8°, strategy 1: 26°, strategy 2: 14°). Furthermore, strategy 3 provided a coating that persisted for up to 8 weeks. Thus, samples obtained with strategy 3 were selected for further tests and comparisons. It is important to underline that all three strategies successfully produced zwitterionic coatings that displayed high hydrophilicity and stability over time. Based on our results, strategy 3 provided the lowest median WCA values and preserved hydrophilicity after 8 weeks of immersion under physiological conditions. Considering that greater coating stability may translate into prolonged activity over time, thereby potentially enhancing the integration of the implanted device^7^. Moreover, surface hydrophilicity has often been associated with a more favorable modulation of the inflammatory cascade and with macrophage polarization toward anti-inflammatory phenotypes^36–38^. Selecting the most hydrophilic strategy for biological evaluation was consistent with the ultimate aim of this study, i.e., to identify coatings capable of modulating inflammatory responses. In addition to performance, strategy 3 also represents a novelty from the methodological point of view. This approach introduces DMAPAA as a co-monomer in the dopamine/zwitterion co-deposition system, through the participation of the acrylamide group in the radical copolymerization with dopamine-derived intermediates and zwitterionic methacrylates, thereby increasing the stability of the poly-zwitterionic chains (Supplementary Fig. S4). Furthermore, the presence of a tertiary amine group contributes to enhanced surface hydrophilicity, as suggested by studies on related acrylamides such as DMAPAA, which has been reported to act as a stabilizer and to improve hydrophilic properties in polymer networks^35^. Thus, based on the above considerations, our biological assays were focused on strategy 3: this strategy provided the most hydrophilic and stable coatings among the three, and it introduced an innovative chemical component (DMAPAA) that has not previously been applied to PEEK functionalization.

**Fig. 4:**
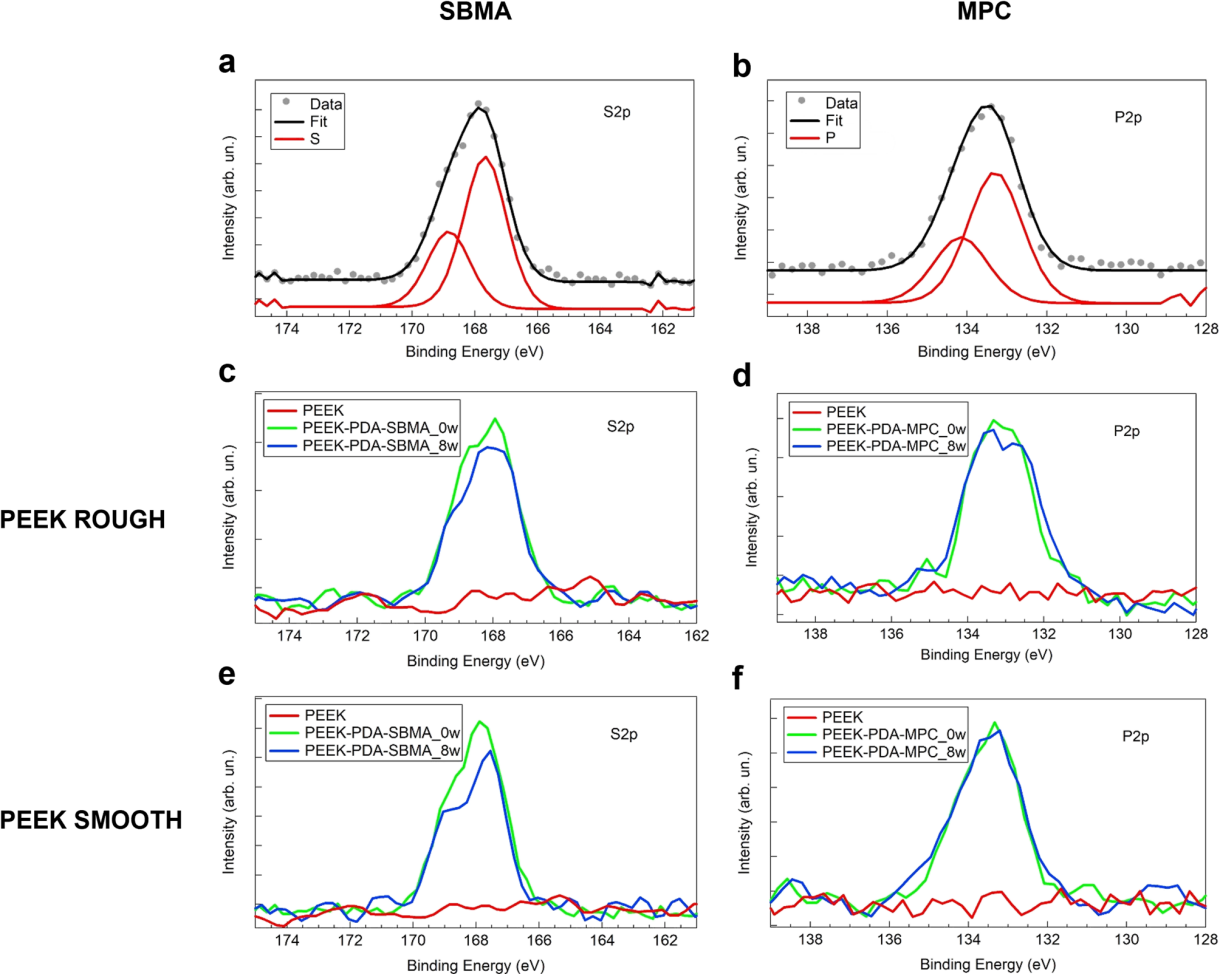
XPS analysis of (left column) the S2p core level region of SBMA and (right column) the P2p core level region of MPC. Deconvolution of (**a**) S2p and (**b**) P2p regions of typical fresh films of SBMA and MPC, respectively. Intensity evolution of the S2p signal for SBMA film on (**c**) ROUGH and (**e**) SMOOTH PEEK, and the P2p signal for MPC film on (**d**) ROUGH and (**f**) SMOOTH PEEK. PEEK = polyether-ether-ketone, SBMA = [2-(methacryloyloxy)ethyl]dimethyl-(3-sulfopropyl)ammonium hydroxide, MPC = 2-methacryloyloxyethyl phosphorylcholine.

To further confirm the stability of zwitterionic coatings obtained with strategy 3, XPS analysis was performed on fresh samples and on samples kept for 8 weeks in physiological conditions. Sulfur and phosphorus were chosen as the characteristic elements for SBMA and MPC, respectively. Figure 4a and b report representative high-resolution spectra of S2p and P2p core level regions of fresh films of SBMA and MPC, respectively.

The S2p core level region (Fig. 4a) can be deconvoluted using a single doublet, with a binding energy of the main 2p3/2 component at 167.7 eV ± 0.2 eV. This signal can be attributed to the presence of the sulfonate group (-SO_3_^−^) of the SBMA molecules. The P2p core level region (Fig. 4b) can be deconvoluted using a single doublet, with a binding energy of the main 2p3/2 component at 133.3 eV ± 0.2 eV. The position of the spectrum is in agreement with the presence of the phosphate group (PO_4_^3−^) of the MPC molecules^49^. The above-reported spectra deconvolution with the same binding energy positions of the 2p3/2 components holds for the spectra reported in Fig. 4c–f, for both 0w and 8w samples. For clarity in reading spectra, only raw data are reported. Figures 4 show the sulfur (4c for ROUGH surface and 4e for SMOOTH surface) and phosphorus (4d for ROUGH surface and 4f for SMOOTH surface) core level regions of samples 0w (green line) and 8w (blue line). The red line in each panel shows the XPS signal of the same core level region acquired on the bare PEEK surface before the deposition of the zwitterionic film and is reported as a reference.

The percentage of sulfur and phosphorus of 0w and 8w samples was derived from the analysis of the S2p and P2p high-resolution XPS spectra. The results indicate that the zwitterionic films are characterized by good stability after 8 weeks of storage at 37 °C under continuous shaking. In particular, the sulfur percentage on SBMA coatings changes from 0.7 ± 0.1 (at 0w) to 0.6 ± 0.1 (at 8w) for ROUGH surfaces and from 0.8 ± 0.1 (at 0w) to 0.6 ± 0.1 (at 8w) for SMOOTH surfaces, showing no significant variation within experimental uncertainty.

As concerns the phosphorus percentage of MPC coatings, values change from 1.0 ± 0.1 (at 0w) to 1.1 ± 0.1 (at 8w) for ROUGH surfaces and from 1.1 ± 0.1 (at 0w) to 1.0 ± 0.1 (at 8w) for SMOOTH surfaces, with no significant variation within experimental uncertainty.

To further characterize the coatings obtained with strategy 3, focused ion beam-scanning electron microscopy (FIB-SEM) imaging was performed on selected samples to directly visualize and measure the thickness of the deposited films. While wettability and surface chemical composition provide indirect confirmation of the coating’s presence and composition, FIB-SEM imaging of sample cross sections enables direct morphological assessment, offering valuable insights into the coating’s uniformity and spatial distribution across the PEEK substrate.

**Fig. 5:**
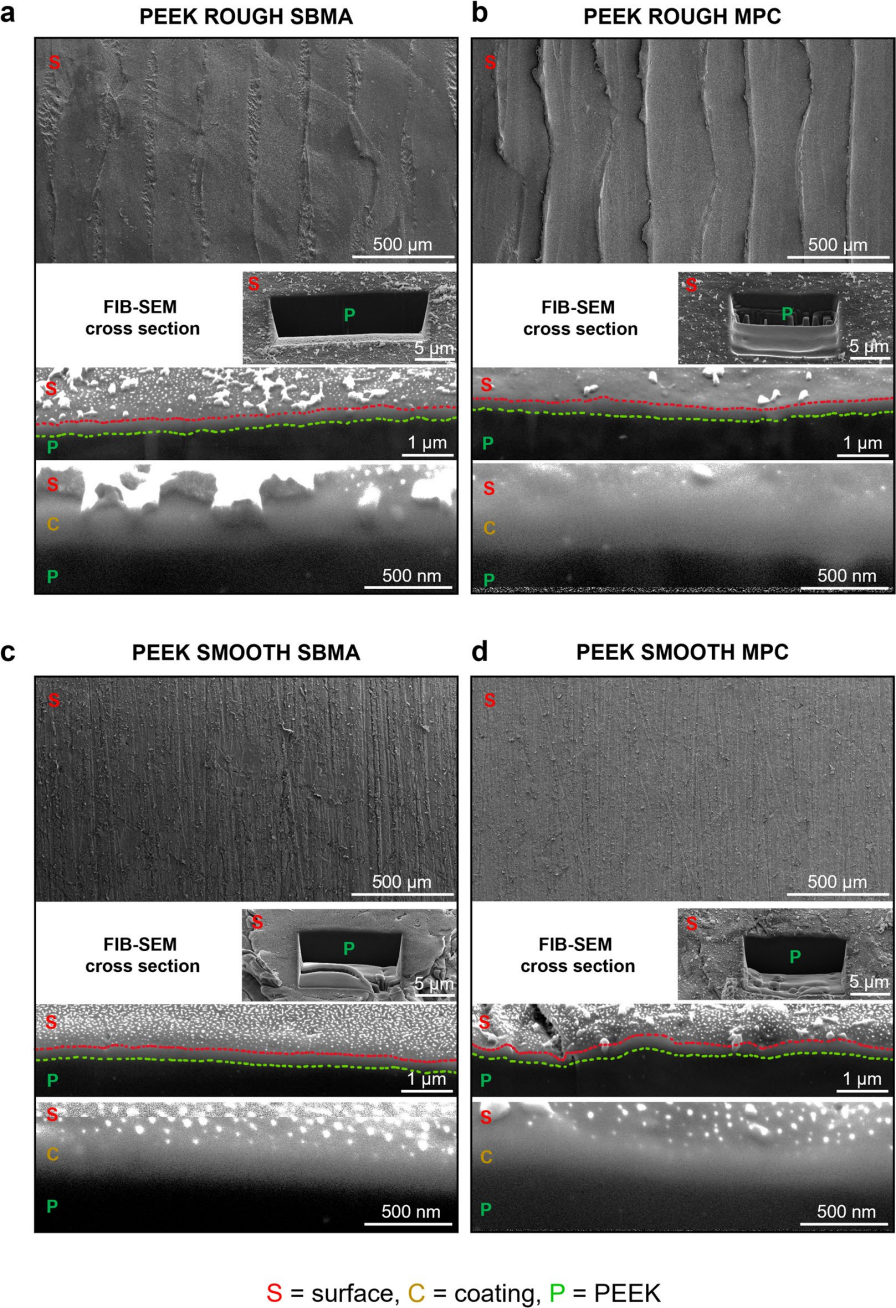
FIB-SEM images of (left column) samples with SBMA-based coating and (right column) samples with MPC-based coating fabricated with the third strategy. In each subfigure, from the top to the bottom, SEM images and FIB-SEM cross-section images at different magnifications are reported: scale bar 5 μm; scale bar 1 μm and coating visualization (between line red and line green); scale bar 500 nm and coating visualization (marked with letter C). (**a**) PEEK ROUGH functionalized with SBMA-based coating. (**b**) PEEK ROUGH functionalized with MPC-based coating. (**c**) PEEK SMOOTH functionalized with SBMA-based coating. (**d**) PEEK SMOOTH functionalized with MPC-based coating. FIB-SEM = focused ion beam-scanning electron microscopy, PEEK = polyether-ether-ketone, SBMA = [2-(methacryloyloxy)ethyl]dimethyl-(3-sulfopropyl)ammonium hydroxide, MPC = 2-methacryloyloxyethyl phosphorylcholine.

Cross-sectional images revealed the presence of a continuous coating layer on both PEEK ROUGH (Fig. 5a, b) and PEEK SMOOTH (Fig. 5c, d) substrates for samples functionalized with strategy 3, which was previously selected. As shown in Fig. 5, the coating layer was clearly distinguishable from the underlying PEEK matrix, showing a uniform profile over the entire observed area. The measured average thickness of the coating layer ranged between 150 nm and 250 nm. These values are in line with those reported for polymeric thin films deposited via PDA-mediated strategies on different surfaces^45^ and support the idea of a uniform thin film formation. The FIB-SEM analysis thus confirms that the hydrophilic and chemically stable coatings obtained with strategy 3 consist of a few hundred nanometers-thick homogeneous films, which are well adherent to the PEEK substrate. This structural integrity is a crucial requirement for biomedical applications where prolonged interaction with physiological environments is expected.

#### In vitro tests

Several in vitro tests were carried out to evaluate the effectiveness of coating in modulating cell adhesion and the production of inflammatory markers. A representative scheme of these tests is reported in Supplementary Fig. S3.

#### Cell adhesion

The acute inflammatory state, which follows device implantation, draws cells of the immune system, headed by macrophages that adhere to the surface of the foreign body. The release of cytokines and chemokines recruits fibroblasts at the site of the lesion^5,50,51^. If the chronic inflammatory state following the acute phase is solved, the device integrates with the surrounding tissue, including macrophages and fibroblasts^5,6^. Thus, the adhesion of fibroblasts and macrophages to the substrate is desirable, but it is important to consider reducing the production of pro-inflammatory markers.

**Fig. 6:**
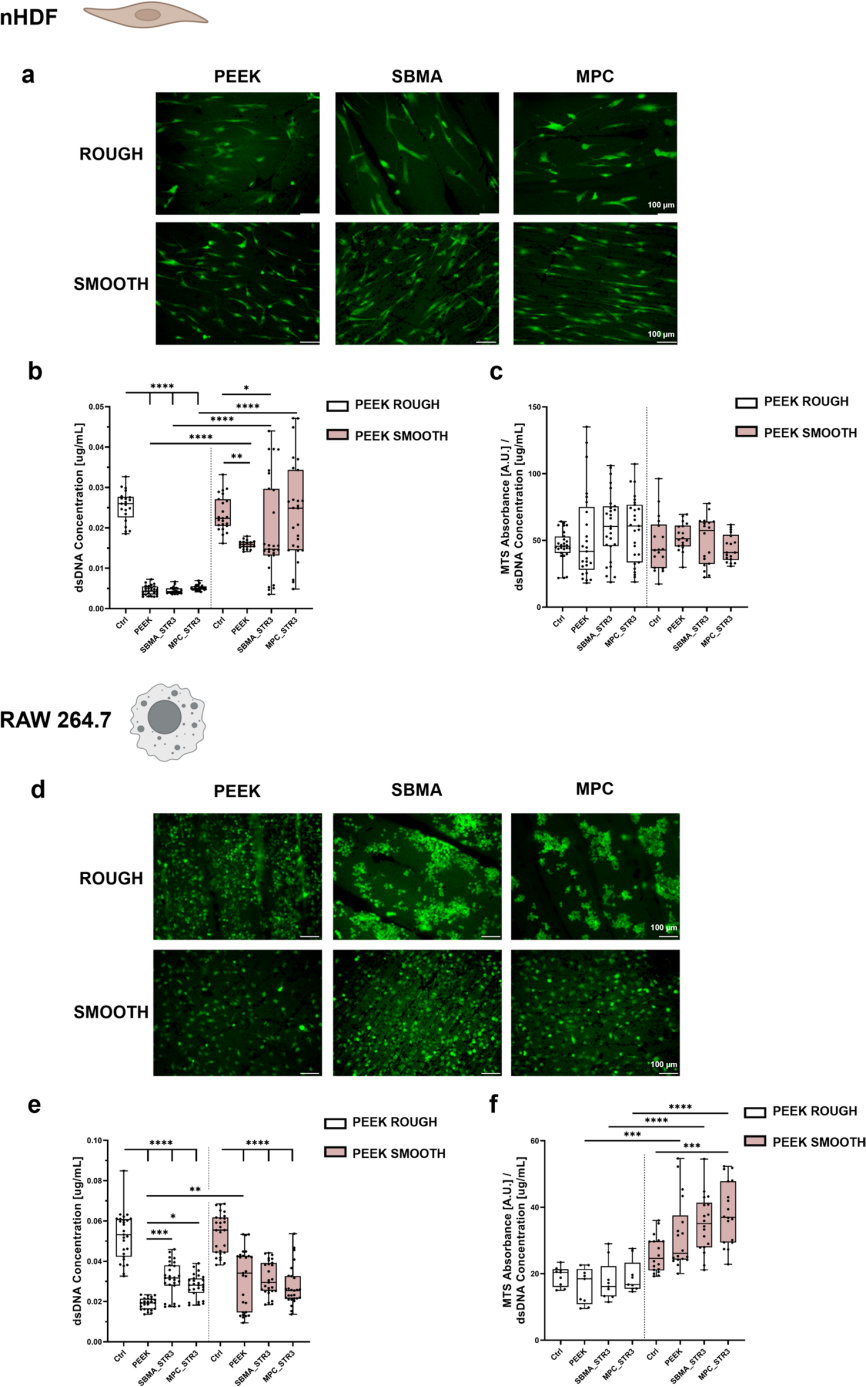
Evaluation of nHDF and RAW 264.7 adhesion on uncoated (PEEK) and coated (SBMA and MPC) samples. Representative LIVE/DEAD images (green: viable cells; scale bar: 100 μm) of (**a**) nHDF and (d) RAW 264.7. Evaluation of cell proliferation of (**b**) nHDF and (**e**) RAW 264.7. Evaluation of cell metabolism of (**c**) nHDF and (**f**) RAW 264.7. * = *p* < 0.05; ** = *p* < 0.01; *** = *p* < 0.001; *** = *p* < 0.0001. *N* = 3. PEEK = polyether-ether-ketone, SBMA = [2-(methacryloyloxy)ethyl]dimethyl-(3-sulfopropyl)ammonium hydroxide, MPC = 2-methacryloyloxyethyl phosphorylcholine, STR3 = strategy 3.

As shown in Fig. 6a, b, fibroblasts adhered better on PEEK SMOOTH than on PEEK ROUGH, and the coatings (obtained through strategy 3) did not affect cell adhesion. Cell viability, evaluated through the MTS assay and normalized to the DNA concentration (Fig. 6c), confirmed that the cells were highly viable on both PEEK ROUGH and SMOOTH, as well as on the substrates coated with the SBMA and MPC coatings.

Regarding the adhesion of RAW264.7 macrophages (Fig. 6d, e), the coatings obtained with strategy 3 on the PEEK ROUGH allowed the cells to adhere to the surface just as they did on the PEEK SMOOTH with and without coatings. The cells showed better viability on PEEK SMOOTH than PEEK ROUGH, especially for substrates functionalized with MPC (Fig. 6f). A better coating distribution, due to the smooth surface, could affect the distribution and viability of cells^52^. In the images (Fig. 6d), it can be observed that cells showed a different distribution on the surfaces of PEEK ROUGH and PEEK SMOOTH, after coating. On PEEK ROUGH, the cells are organized in clusters, probably due to a less uniform distribution of the coating, as mentioned above.

The adhesion of normal human dermal fibroblast (nHDF) and RAW 264.7 macrophage observed on the coated samples is a desirable result, as noted earlier, indicating potential for proper device integration post-implantation, if inflammation is effectively controlled. Our results seem to contradict other reports, in which SBMA and MPC coatings showed strong anti-fouling activity, considerably reducing the adhesion of fibroblasts and macrophages, with respect to non-coated surfaces^11,45,53^. However, these results were obtained by functionalizing different surfaces, such as silicon wafers, polydimethylsiloxane, polyimide, and stainless steel, which showed high cell adhesivity. PEEK, in itself, is featured by a relatively low cell adhesiveness, as shown in Fig. 6b, e, where the difference between the polystyrene well (labeled as Ctrl) and PEEK ROUGH or SMOOTH is statistically different (*p* < 0.0001). The cell adhesion results can thus be rationalized by considering both the intrinsic properties of PEEK and the specific features of the coatings obtained with strategy 3. Fibroblasts and macrophages adhered more efficiently on PEEK SMOOTH than on PEEK ROUGH, a trend preserved after functionalization with SBMA or MPC. On SMOOTH substrates, the coating distribution was more homogeneous, which likely favored a more even cell spreading and higher viability, particularly for MPC. On ROUGH substrates, the less uniform deposition produced patchy coverage and cell clustering, reflecting local variations in protein adsorption and adhesiveness. These observations are consistent with the literature showing that microscale uniformity and surface continuity are critical determinants of cell morphology and signaling^54^. From a biological perspective, the preserved adhesion of fibroblasts and macrophages on zwitterionic-coated PEEK is advantageous. Successful integration of an implant requires that immune cells establish contact with the material surface, while excessive inflammatory activation must be minimized^55^. Importantly, cell adhesion is tightly linked to the underlying metabolic state: the higher adhesion and viability of macrophages on PEEK SMOOTH can be explained by the more uniform distribution of the zwitterionic coating on smooth substrates. A homogeneous and continuous hydration layer provides a consistent set of physicochemical cues, which allows macrophages to adhere in a more stable and organized manner. In contrast, the heterogeneous coverage typically observed on PEEK ROUGH generates local discontinuities in surface chemistry and protein adsorption, leading to clustered adhesion and uneven spreading. These conditions are known to reinforce glycolytic, pro-inflammatory metabolism in macrophages, limiting their viability and promoting the secretion of inflammatory mediators^56–58^. Conversely, uniform and hydrophilic coating on PEEK SMOOTH surfaces favors balanced integrin engagement and cytoskeletal organization. This mechanistic link between coating homogeneity, adhesion pattern, and macrophage metabolism provides a rationale for the improved cellular responses observed on SMOOTH compared with PEEK ROUGH samples. Similarly, fibroblasts adhering to smoother and more uniformly coated PEEK may benefit from improved cytoskeletal organization and mitochondrial activity, supporting viability without excessive proliferation. The apparent discrepancy with reports of reduced adhesion on zwitterionic surfaces^59,60^ is therefore explained by differences in substrate type, coating strategy, and baseline adhesiveness: our novel co-deposition approach on PEEK yields surfaces that retain sufficient adhesiveness for physiological integration, while conferring a significant immunomodulatory effect.

#### Evaluation of inflammatory markers

As mentioned, modulating the inflammatory response is crucial for the effective integration of an implanted device. The release of the main pro-inflammatory cytokines by macrophages was detected to assess the ability of the zwitterionic coatings to modulate them.

**Fig. 7:**
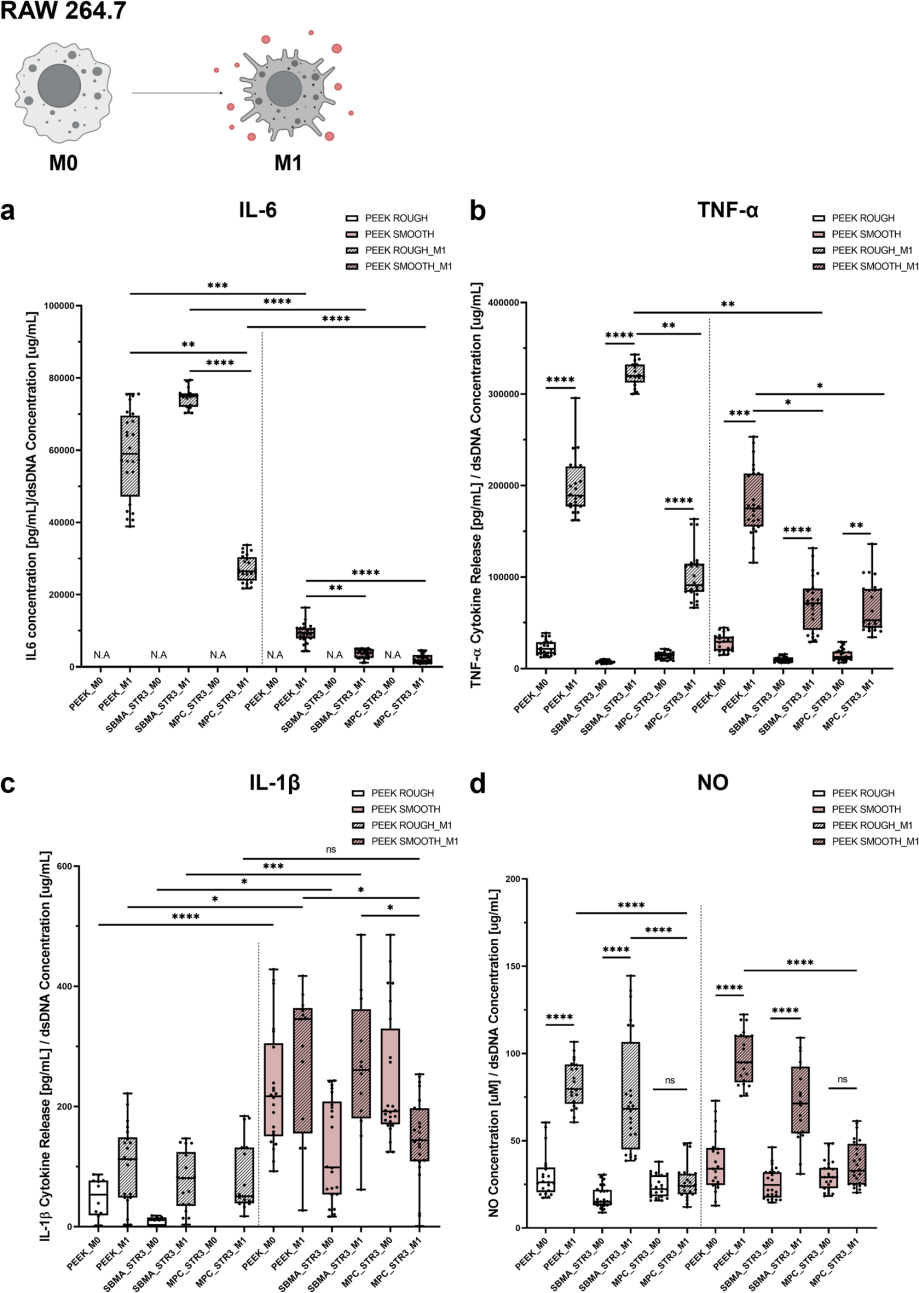
Pro-inflammatory cytokine and NO release. Evaluation of (**a**) IL-6, (**b**) TNF-α, (**c**) IL-1β and (**d**) NO for PEEK ROUGH and PEEK SMOOTH surfaces. * = *p* < 0.05; ** = *p* < 0.01; *** = *p* < 0.001; *** = *p* < 0.0001. Biological replicates = 4. N/A = not applicable. PEEK = polyether-ether-ketone, SBMA = [2-(methacryloyloxy)ethyl]dimethyl-(3-sulfopropyl)ammonium hydroxide, MPC = 2-methacryloyloxyethyl phosphorylcholine, STR3 = strategy 3, M0 = RAW 264.7 macrophage, M1 = RAW 2647 macrophage skewed towards pro-inflammatory phenotype.

In the graphs of Fig. 7, samples labeled with “M0” refer to macrophage culture with the M0 phenotype, while samples labeled with “M1” refer to macrophages skewed toward an M1-like phenotype (pro-inflammatory state) by adding lipopolysaccharide (LPS) in the culture media.

In Fig. 7a, the release of IL-6 is shown. Without treatment with LPS (M0 samples), IL-6 could not be detected, while it became significant after stimulation of cells with LPS (M1 samples). The release of IL-6 was lower for the PEEK SMOOTH samples than the PEEK ROUGH samples. This was probably due to the different roughness, which is known to influence considerably the production of IL-6^61,62^. MPC-based coatings (reported in graphs as MPC_STR3_M1) reduced the IL-6 release on both ROUGH and SMOOTH substrates, considerably, even if a greater effect was found on PEEK SMOOTH (*p* < 0.0001 vs. *p* < 0.01 for PEEK ROUGH). Instead, SBMA-based coatings (reported in graphs as SBMA_STR3_M1) reduced the release of IL-6 only on PEEK SMOOTH (*p* < 0.01).

Regarding TNF-α (Fig. 7b), for PEEK ROUGH, MPC coatings (reported in the graphs as MPC_STR3_M1) were much more effective than SBMA ones (reported in the graphs as SBMA_STR3_M1), in reducing the production of this cytokine. For PEEK SMOOTH, a reduction in TNF-α release was observed on all coated samples (both for SBMA- and MPC-based coatings, *p* < 0.1). Comparing the MPC-based coatings on the different substrates, the median value obtained on the SMOOTH samples was lower than that obtained on the ROUGH samples.

As shown in Fig. 7c, no significant differences were found between coated and non-coated samples concerning the release of IL-1β. In general, the PEEK ROUGH samples, with and without coatings, showed smaller values than the PEEK SMOOTH samples. The only exception was found for MPC-based coatings on PEEK SMOOTH, which kept the release of IL-1β at values comparable with the ones featuring PEEK ROUGH samples (reported in the graphs as MPC_STR3_M1).

Finally, the release of nitric oxide (NO) is shown in Fig. 7d. Samples provided with SBMA-based coatings (reported in the graphs as SBMA_STR3_M1) showed similar values to non-coated PEEK samples (reported in the graphs as PEEK_M1). Instead, remarkably, MPC-based coatings (reported in the graphs as MPC_STR3_M1) considerably reduced NO levels, bringing them to values similar to the basal ones, featuring the M0 samples (reported in the graphs as MPC_STR3_M0).

Macrophage activation is highly sensitive to nature, conformation, and physicochemical cues from the substrate (wettability, chemistry, local stiffness, and topography). Our data shows a consistent reduction in pro-inflammatory mediators (IL-6, TNF-α, NO) for MPC_STR3 on PEEK SMOOTH (Fig. 7). This effect can be explained by two synergistic contributions: (i) the zwitterionic hydration layer reduces deposition and favors the denaturation of pro-inflammatory proteins, lowering receptor-mediated activation of macrophages^63^; and (ii) the uniform and stable presence of zwitterionic groups reduces local heterogeneities that can act as focal activation sites. Importantly, although cell adhesion was not dramatically reduced on coated PEEK (likely because baseline PEEK adhesiveness is already low), the qualitative adhesion pattern (clusters on ROUGH; more evenly spread cells on SMOOTH) indicates that local coating distribution and topography still modulate cell morphology and local cell-cell signaling, which are known to influence macrophage phenotype and cytokine secretion^58^.

The reduction of pro-inflammatory mediators observed on MPC_STR3 surfaces, particularly on PEEK SMOOTH, can be interpreted in light of well-established immunological mechanisms. Macrophage activation is highly sensitive to the composition and conformation of the adsorbed protein layer, which in turn is dictated by surface chemistry and hydrophilicity. Zwitterionic coatings, and especially MPC with its phosphorylcholine group, generate a dense hydration shell that resists non-specific protein adsorption and prevents conformational changes of key proteins^64^. This effect limits the engagement of Toll-like receptors (TLRs) and integrins, thereby dampening Nuclear factor-κB (NF-κB) activation and reducing downstream cytokine expression. Consistently, IL-6 and TNF-α, two prototypical NF-κB–driven cytokines^65^, were significantly decreased on MPC_STR3, in line with previous reports showing that hydrated zwitterionic interfaces attenuate macrophage inflammatory signaling^28,66^. NO, produced by iNOS as a hallmark of M1 polarization^67^, was also strongly reduced, reflecting the same suppression of NF-κB–mediated transcription and possibly a metabolic shift toward arginase pathways, which divert arginine from NO synthesis to tissue-repair routes^68^. By contrast, IL-1β secretion showed limited changes across conditions. This is consistent with its biology, since IL-1β release requires not only transcriptional priming (achieved here by LPS) but also a second inflammasome-activating signal^69^, which was not modulated by the coatings in our setup. Finally, the comparison between ROUGH and SMOOTH substrates highlights the importance of topography: ROUGH PEEK may hinder uniform deposition and create local undercoated areas that act as focal activation sites, whereas SMOOTH PEEK favors homogeneous coating growth and a continuous hydration barrier, amplifying the immunomodulatory effect. Altogether, these results indicate that the improved hydrophilicity and homogeneity of MPC_STR3 coatings reduce the adsorption of pro-inflammatory proteins and blunt receptor-mediated macrophage activation, thereby explaining the coherent decrease in IL-6, TNF-α and NO, while leaving IL-1β largely unaffected.

In the state of the art, zwitterionic coatings have shown the ability to modulate the inflammatory response of macrophages^28,45^. However, a systematic comparison between SBMA and MPC on PEEK substrates was not available. We clarified for the first time the effects of these two zwitterions on the production of pro-inflammatory markers by macrophages, on PEEK surfaces provided with different surface roughnesses. It should be noted that this study focused on the modulation of pro-inflammatory responses (IL-1β, IL-6, TNF-α, and NO), while not assessing anti-inflammatory cytokines such as interleukin-4 (IL-4) and interleukin-10 (IL-10), which are typically associated with macrophage polarization toward the M2 phenotype. Although our findings suggest a shift toward a less inflammatory environment, we cannot conclude that SBMA- and MPC-modified surfaces directly promote M2 polarization. Future in vitro and in vivo studies will specifically investigate the expression of M2-associated cytokines (IL-4, IL-10) and phenotypic markers (e.g., CD206, Arg1) to elucidate more in-depth the immunomodulatory potential of these coatings.

## Conclusion

In this paper, we compared three coating strategies for introducing zwitterionic molecules onto the PEEK surface, with two roughness levels (PEEK ROUGH and PEEK SMOOTH). For all strategies, PDA was used to facilitate the functionalization of PEEK with SBMA or MPC zwitterionic molecules. The first strategy involved depositing a PDA layer, followed by functionalization with a zwitterionic monomeric layer. The second and third strategies co-deposited dopamine and zwitterionic monomers to form a zwitterionic polymer layer, with strategy 3 using DMAPAA monomers to create a zwitterionic copolymer layer. The coatings demonstrated high hydrophilicity. Strategy 3 consistently provided the highest hydrophilicity, with a median(IQR) WCA of 8°(7–11), and showed excellent stability, maintaining its coating for up to 8 weeks under physiological conditions. Finally, the in vitro evaluations were performed only on coatings obtained using strategy 3, which had previously demonstrated superior hydrophilicity and long-term stability, a desirable aspect for modulating the inflammatory response. These biological tests confirmed the ability of strategy 3 coatings (especially those fabricated on PEEK SMOOTH) to modulate inflammatory responses, as evidenced by improved macrophage viability and reduced release of pro-inflammatory cytokines. Furthermore, the MPC-based coating appeared to be more promising than the SBMA-based coating, inhibiting nitric oxide release. These findings underscore the potential of these coatings to not only improve biocompatibility but also enhance the functional integration of PEEK implants, suggesting a valuable approach for reducing inflammation and promoting tissue healing post-implantation. Future in vivo studies are needed to validate these results, providing a deeper understanding of their long-term impact on implant safety, durability, and overall performance in clinical applications. The use of DMAPAA monomers in strategy 3 is also promising in terms of potential antimicrobial effects^70^, to be investigated in future studies.

## Materials and methods

### Fabrication of PEEK substrates

In this study, 3D printed PEEK samples (Fig. 2a) with a diameter of 12 mm and a height of 1 mm were used. Two different surface finishes, which were named ROUGH and SMOOTH, were compared. First, a virtual model of the disc was designed using 3D software (SolidWorks 2023) and exported as a Standard Tesselation Language (STL) file. Both surfaces were then produced by Roboze Spa (Italy), starting from the STL file and using their Roboze ARGO 500 3D printer, which is based on FDM technology optimized for high-performance materials. The samples were printed using a 1.75 ± 0.05 mm diameter PEEK filament (Roboze spa, Italy).

As reported by the supplier, before printing, the PEEK spools were subjected to a drying cycle at 100 °C for 12 h in an HT Dryer, to minimize the concentration of water molecules adsorbed by the filament due to exposure to the atmospheric environment. The printing parameters, namely printing speed, nozzle diameter, extrusion temperature, bed temperature, chamber temperature, layer thickness, and infill percentage, were set at 1200 mm/min, 0.6 mm, 420 °C, 150 °C, 160 °C, 0.2 mm, and 100%, respectively. To ensure isothermic conditions within the hot chamber, a 2-hour stabilization delay was applied before starting the process.

The PEEK ROUGH discs are discs in their as-printed state. The post-processing consisted only of support removal, followed by general cleaning with a cutting tool, without any additional surface treatment or finishing. These samples, therefore, represent the untreated condition of additive manufacturing.

The PEEK SMOOTH discs underwent a manual multi-stage post-processing procedure aimed at achieving a visibly smoother surface. After support removal, a sanding procedure was performed. Given the small dimensions of the discs, a custom 3D-printed tool was designed to fix the samples in place during sanding, ensuring stability, greater precision, and improved uniformity of the process. The sanding was performed in multiple stages, using sandpapers with progressively finer grit sizes, each applied for about 15 min. Initially, P120 grit sandpaper was used to remove surface imperfections and obtain a preliminary level of roughness. This was followed by P220 and P320 grit sandpaper to refine the surface, further reducing the roughness and improving the uniformity of the texture. Finally, the discs were treated with P400 grit sandpaper to achieve a visually homogeneous surface. The progressive use of different grits allowed a gradual reduction in surface roughness.

The final roughness was then quantified and validated by optical profilometry analysis (Fig. 1b), as described in the following section, to demonstrate a statistically significant difference between the ROUGH and SMOOTH samples.

### Characterization of PEEK surfaces

To characterize the surfaces before coating them, SEM (Phenom XL, Thermo Fisher, USA, beam voltage: 10 kV, under vacuum conditions) imaging, static WCA (contact angle goniometer, Ossila Ltd., UK) measurements, and optical profilometry (Zeiss LSM 900 confocal laser scanning microscope, Carl Zeiss Microscopy GmbH, Germany) analysis were carried out^21^ (Fig. 1). To appreciate the different appearance of the two surfaces, SEM images were taken, using two different magnifications (180X and 10000X). Then, a surface wettability analysis was done. A total of fifty-eight discs of PEEK ROUGH and sixty discs of PEEK SMOOTH were subjected to static WCA measurements. A 7.5 µL droplet of deionized water was placed on the samples’ surface and, thanks to an incorporated camera, a digital photo was taken. The resulting contact angle was evaluated from the software provided by the company. For each sample, three different measurements were performed, testing a different point on the surface. Then, the average value was registered.

The surfaces of PEEK ROUGH and PEEK SMOOTH (three samples each) were reconstructed both in 2D and 3D using a non-contact optical profilometer. Analyses were carried out on five different points per sample; both peripheral and central areas were investigated. A representative image for each surface was selected, and the Sa values were reported and compared.

### Coating fabrication

Three strategies were used to functionalize the PEEK surfaces (Fig. 2b). For each strategy, ten independent samples, both for ROUGH and SMOOTH surfaces, were used (except for the first strategy with SBMA on PEEK ROUGH, where eight samples were used). They were cleaned by soaking them in a 70% v/v ethanol solution and ultrasonicating them for 15 min^21^. Then, they were left to dry overnight.

Strategy 1 (Fig. 2b) consisted of two steps. In the first one, a polydopamine layer was obtained on the substrates (PEEK-PDA). The samples were immersed for 24 h at room temperature (r.t.) in a solution of 10 mM Tris buffer (pH 8.5, Sigma-Aldrich, 88438) containing dopamine hydrochloride (Sigma-Aldrich, H8502, Fig. 2a) at a concentration of 2 mg/mL^44^. After 24 h, the samples were gently rinsed with deionized water and left to dry overnight^29^. In the second step, the PEEK-PDA substrates were functionalized with a zwitterionic monolayer via Michael addition, where the acrylate moiety of the zwitterions reacts with the amine group of PDA to form a β-amino linkage (Supplementary Fig. S4)^15,44^. A reaction solution of deionized water, ethanol, and triethylamine (TEA), in a volume ratio of 400:80:3, was prepared and enriched with the zwitterionic monomers (SBMA, Sigma-Aldrich, 537284 or MPC, Sigma-Aldrich, 730114 Fig. 2a) at a concentration of 100 mM. Then, the coated samples, thanks to an ad-hoc structure, were immersed in the reaction solution and left to be magnetically stirred at r.t. for 24 h^71^. Finally, they were rinsed with deionized water and stored in a 1X PBS solution (Sigma Aldrich, P4417).

Strategy 2 and strategy 3 are both one-step processes, where the co-deposition of dopamine and zwitterions was performed. During this process, the spontaneous auto-oxidation of dopamine generates radicals that initiate the free radical polymerization of zwitterions, resulting in covalent bonding with the benzene ring of dopamine (Supplementary Fig. S4)^47^. In detail, for strategy 2 (Fig. 2b), the cleaned samples were first pre-wetted in a solution of 100% ethanol and then immersed in a different reaction solution composed of 50 mM Tris buffer solution (pH 8.5), dopamine hydrochloride, and zwitterionic monomers (SBMA or MPC) with a mass ratio of 1:15 ^47^. A magnetic stirrer was used to maintain the solution in agitation for 8 h at r.t., then the samples were soaked in deionized water and left there overnight^47^, before being stored in 1X PBS solution.

Strategy 3 (Fig. 2b) was a novel strategy for the deposition of dopamine and a co-polymer made from the zwitterionic and DMAPAA monomers (AmBeed, A329359 Fig. 2a). The cleaned samples were immersed in the same solution of strategy 2 enriched with DMAPAA at a concentration of 0.3 mg/mL.

In all three strategies, the surface modification is physically mediated by self-polymerization of dopamine, which generates a stable PDA layer that strongly adheres to PEEK. This adhesive layer provides catechol and amine groups that can be further functionalized with SBMA or MPC, ensuring long-term coating stability^30^.

### Coating characterization and stability tests

The presence of the coatings (zwitterionic coatings mediated by PDA) was first evaluated through an indirect method^47,53,72^. As shown in the state-of-the-art, the PDA and zwitterionic layers feature high hydrophilicity^15,43^. Thus, surface wettability before and after the coating fabrication was investigated through WCA measurements. Three different measurements were made for each sample, placing 7.5 µL of deionized water on the surface. The average value of these measurements was taken into account.

A stability test was carried out. After modification with a zwitterionic coating, all samples were kept in an environment mimicking physiological conditions for 8 weeks. The samples were soaked in 1X PBS, maintained under continuous shaking (200 rpm) using an orbital shaker (Mod. 7114/CT, ASAL s.r.l.), and kept at a controlled temperature of 37 °C. To verify coating stability, WCA measurements were performed and compared at different time points: 1 week, 2 weeks, 3 weeks, 4 weeks, and 8 weeks.

XPS measurements were also performed to confirm the presence of zwitterionic coatings. Surface chemical analysis was carried out using a PHI 5600 Multi-Technique XPS apparatus (Chanhassen, MN, USA). The X-rays were generated with an Al-monochromatized source (hυ = 1486.6 eV), and a low-energy electron flood gun (neutralizer) was used to avoid sample charging during measurements^73^. A pass energy of 29.35 eV was used for high-resolution spectra acquisition. CasaXPS software (v2.3.25PR1.0, Casa Software Ltd., Teignmouth, UK) was used for spectra analysis. Spectra were calibrated by adjusting the carbon adventitious component of the C1s region at a Binding Energy of 284.8 eV. The deconvolution of spectra was performed using 30% Gaussian Voigt functions after a Shirley background subtraction^74^. For the P2p and S2p doublets deconvolution, a spin-orbit splitting of 0.86 eV and 1.16 eV was used, with a 2:1 area ratio between the main and the secondary component^75^.

In order to assess coatings thickness and morphology, PEEK SMOOTH and PEEK ROUGH samples functionalized using strategy 3 with SBMA or MPC were sputter coated with a thin layer of Au/Pd, using a Quorum SC7620 sputter coater (Quorum Technologies Ltd, Lewes, UK), then analyzed using a Helios Nano Lab 600i FIB-SEM (Thermo Fisher Scientific, Waltham, MA, USA). Cross sections of the materials were obtained using the gallium beam impinging perpendicularly (i.e., sample tilt of 52°) onto specific sample surface areas, to prevent unrestrained coating damage, and operating at 30 kV and 2.5 nA. SEM images of the cross sections were acquired with 5 kV and 43pA using a Everhart-Thornley Detector (ETD), detecting secondary electrons (SE), keeping the sample at 4–4.2 mm working distance and tilt angle of 52°. Coating thickness was estimated from 100k magnification images.

#### In vitro tests

Before starting the in vitro tests, samples were grouped into two families, PEEK ROUGH and PEEK SMOOTH, each divided into subgroups: not treated (without any coatings, named PEEK group), substrates functionalized with a zwitterionic coating based on SBMA (named SBMA group), and substrates functionalized with a zwitterionic coating based on MPC (named MPC group). All samples were subjected to three consecutive washes with sterile 1X PBS without calcium and magnesium (PBS w/o), then they were immersed for 30 min in a solution of sterile 1X PBS w/o containing 1% v/v Penicillin-Streptomycin (P/S, Sigma-Aldrich, P4333). Finally, they were washed with sterile 1X PBS w/o.

#### Cell lines and culture conditions

nHDF (Lonza, CC-2511) and RAW 264.7 (ATCC, TIB-71) cell lines were cultured using a complete growth medium (GM) composed of high-glucose Dulbecco’s Modified Eagle’s Medium (DMEM, Sigma-Aldrich, D6429) supplemented with 10% v/v fetal bovine serum (FBS, Sigma-Aldrich, F0804) and 1% v/v P/S in a 5% carbon dioxide humidified atmosphere at 37 °C.

#### Cell adhesion

For such tests, an additional subgroup was considered, namely the CTRL group, consisting of cells cultured on standard polystyrene surfaces (24-well culture plate, Thermo Scientific, 142475). nHDF cells (15k cells/cm^2^^11^) and RAW264.7 cells (30k cells/cm^2^^11^) were seeded on different surfaces (Supplementary Fig. S3a). Qualitative and quantitative analyses were carried out. After 24 h of incubation, cell viability was qualitatively evaluated with a LIVE/DEAD^®^ Viability/Cytotoxicity assay (Invitrogen, L3224). Briefly, GM was removed, and 1X PBS containing 2 µM calcein-AM was added^21^. After incubation at r.t. for 30 min, cells were washed with 1X PBS and observed under a Leica DMi8 microscope. Three samples for each group were used.

Metabolic activity of cells was detected using a MTS assay (Abcam, ab197010) (Supplementary Fig. S3a). Briefly, 50 µL of the MTS reagent described above was added to each well and incubated for 2 h in standard culture conditions. VICTOR Nivo Multilabel plate reader (PerkinElmer, Waltham, MA, USA) was used to read the absorbance signal at a wavelength of 490 nm. Three samples for each group were used.

The number of cells adhered was quantitatively evaluated by assessing the concentration of double-stranded DNA, through a Quant-iT^TM^PicoGreen^®^dsDNA assay kit (Invitrogen, p11496) (Supplementary Fig. S3a). Briefly, the GM was removed, and three freeze-thaw cycles with 500 µL of nuclease-free water (Sigma-Aldrich, W4502) were implemented to lyse the cells. Then, 50 µl aliquots were transferred to a 96-well black round-bottom polystyrene microplate (Corning, 3792), according to the manufacturer’s instructions. After 10 min of incubation in the dark, the assay was performed, and the emitted fluorescence was read with a VICTOR Nivo Multilabel plate reader (excitation wavelength 480 nm, emission wavelength 535 nm). These results were also used to normalize the metabolic activity of the cells. Three samples for each group were used.

#### Evaluation of inflammatory markers

To evaluate in vitro the ability of the coatings to modulate macrophage inflammatory response, the release of pro-inflammatory markers was assessed. The samples were divided into two main families represented by the substrate type, PEEK ROUGH and PEEK SMOOTH; each of them was composed of (i) PEEK discs not treated, (ii) PEEK discs with a zwitterionic coating based on SBMA, (iii) PEEK discs with a zwitterionic coating based on MPC, both fabricated with the third strategy of coating. For all these categories mentioned above, either M0 or M1 macrophages were seeded, for a total of six experimental conditions per substrate type (Supplementary Fig. S3b).

Briefly, RAW 264.7 cells were seeded with a density of 80k cells/cm^2^^76^. After 12 h of incubation, GM was changed for all the samples. Only for M1 groups, cells were stimulated with LPS (from Escherichia coli O111:B4, Sigma-Aldrich, L2630) at a concentration of 1 µg/ml for 24 h, a well-established protocol for M1 polarization in the current literature^76^. At the end, supernatants were collected. Pro-inflammatory cytokine production was analyzed through Mouse IL-1β ELISA kit (Sigma-Aldrich, RAB0274), Mouse IL-6 ELISA Kit (Sigma-Aldrich, RAB0308), and Mouse TNF-α ELISA Kit (Sigma-Aldrich, RAB0477) following the manufacturer’s instructions. The absorbance signal was read with a VICTOR Nivo Multilabel plate reader, setting a primary wavelength of 450 nm for all the kits. Results were converted to numeric values using standard curves. NO production was evaluated through the Griess Reagent kit (Invitrogen, G7921). The absorbance signal was read with a VICTOR Nivo Multilabel plate reader, setting a primary wavelength of 548 nm^11,45,76^. All protein concentration values were normalized to the respective dsDNA concentration to compare samples of different groups. Four samples for each group were used.

#### Statistical analyses

Surface characterization data were normally distributed and presented as mean ± std with bar plots. Statistical significance was assessed using the Unpaired t-test, with a significance threshold of *p* = 0.05. Coating characterization and in vitro test data were non-parametric, as the normality assumption was rejected. These data are shown with violin plots or boxplots, displaying the median and 25th–75th quartiles. Kruskal–Wallis with Dunn’s post-hoc test was used to identify statistically significant differences, with a significance threshold of *p* = 0.05. All analyses were performed using GraphPad Prism 9 (GraphPad Software Inc.).

## Supplementary Information

Below is the link to the electronic supplementary material.

Supplementary Material 1

## Data Availability

The data that support the findings of this study are available from the corresponding author upon reasonable request.

## Abbreviations

PEEK: Polyether-ether-ketone
MPC: 2-methacryloyloxyethyl phosphorylcholine
SBMA: [2-(methacryloyloxy)ethyl]dimethyl-(3-sulfopropyl)ammonium hydroxide
FBR: Foreign body reaction
TNF-α: Tumor necrosis factor-α
IL-6: Interleukin-6
IL-β: Interleukin-β
PEG: Polyethylene glycol
FDM: Fused deposition modeling
WCA: Water contact angle
SEM: Scanning electron microscopy
PDA: Polydopamine
XPS: X-ray photoelectron spectroscopy
DMAPAA: N-[3-(dimethylamino)propyl]acrylamide
IQR: Interquartile range
FIB-SEM: Focused ion beam-scanning electron microscopy
PBS: Phosphate buffer saline
nHDF: Normal human dermal fibroblast
LPS: Lipopolysaccharide
STR3: Strategy 3
NO: Nitric oxide
r.t.: Room temperature
TEA: Triethylamine
GM: Growth medium
