# Supplementary material for: Comparison between SBMA and MPC coatings on PEEK surface: stability over time and anti-inflammatory effects in vitr﻿o
Source: Sci Rep. 2025 Nov 21;15:41263. doi: 10.1038/s41598-025-25082-5 (PMC12639175; doi:10.1038/s41598-025-25082-5)
Supplement: Supplementary file 1 — Supplementary Material 1 [file 41598_2025_25082_MOESM1_ESM.doc]

**Supporting Information**

Comparison between SBMA and MPC coatings on PEEK surface: stability over time and anti-inflammatory effects in vitro

Erika Roventini* ^1,2^, Francesco Iacoponi ^1,2^, Aliria Poliziani ^1,2^, Paolo Canepa ^3^, Ornella Cavalleri ^3^, Maria A. Cassa ^4,5^, Paola Parlanti ^6^, Carlotta Pucci ^1,2^, Mauro Gemmi ^6^, Chiara Tonda Turo ^4,5^, Leonardo Ricotti ^1,2^

1 – The BioRobotics Institute, Scuola Superiore Sant’Anna, Piazza Martiri della Libertà 33, 56127 Pisa, Italy

2 – Department of Excellence in Robotics & AI, Scuola Superiore Sant’Anna, Piazza Martiri della Libertà 33, 56127 Pisa, Italy

3 – Department of Physics, University of Genoa, 16146 Genoa, Italy

4 – Department of Mechanical and Aerospace Engineering, Politecnico Di Torino, 10129 Turin, Italy

5 – PolitoBIOMed Lab, Politecnico Di Torino, 10129 Turin, Italy.

6 – Istituto Italiano di Tecnologia, Center for Materials Interfaces, Electron Crystallography, Viale Rinaldo Piaggio 34, 56025 Pontedera, Italy

AUTHOR INFORMATION

Corresponding Author

* Erika Roventini

The BioRobotics Institute, Scuola Superiore Sant’Anna

Viale Rinaldo Piaggio 34, 56025 – Pontedera (PI), Italy

e-mail: [erika.roventini@santannapisa.it](mailto:erika.roventini@santannapisa.it)

ORCID
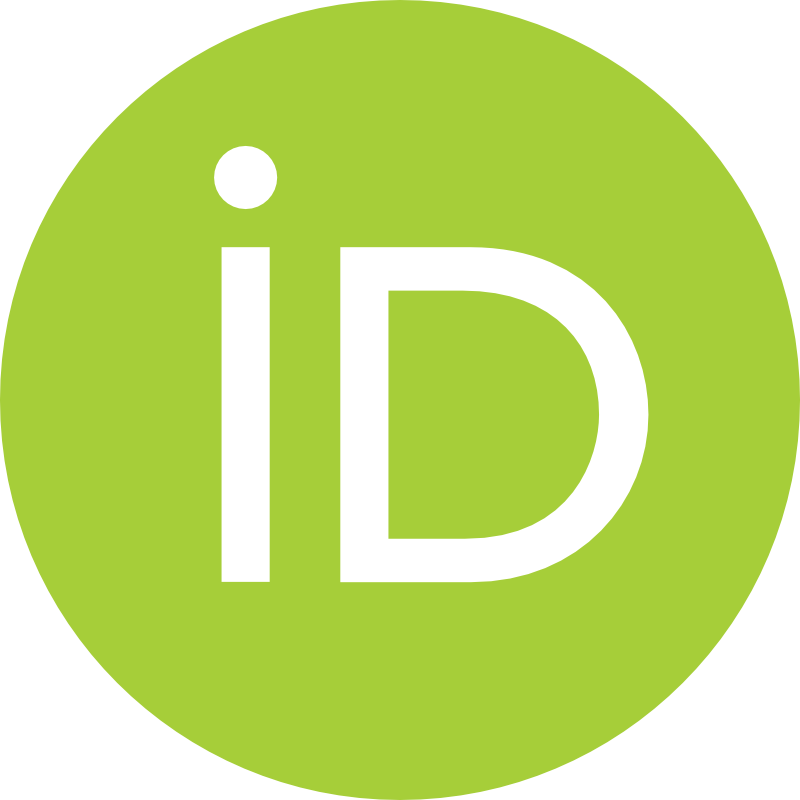
 : <https://orcid.org/0009-0005-2337-1968>


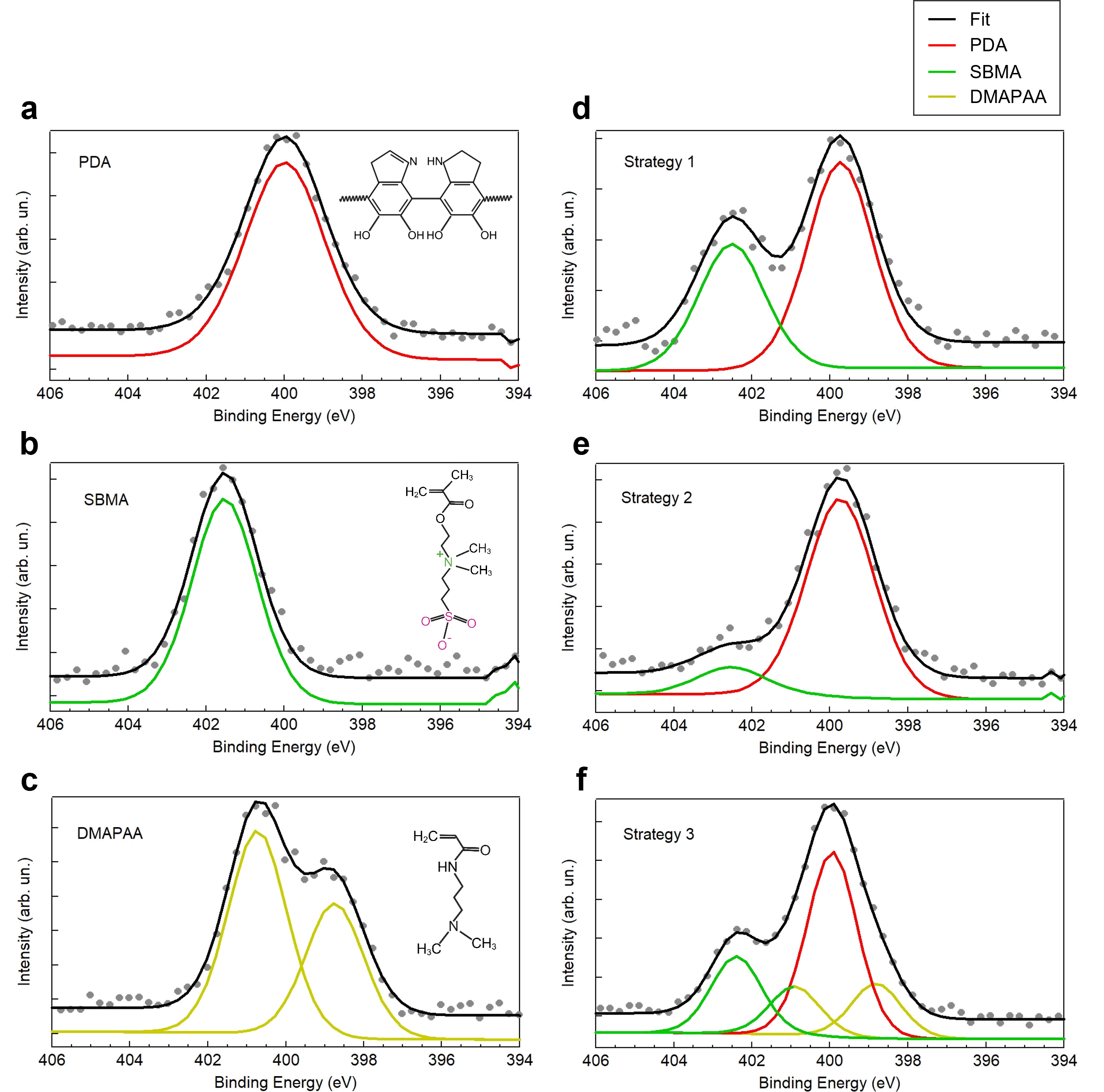


**Supplementary Figure S1.** XPS analysis of nitrogen signals confirms the presence of the SBMA-based coatings obtained with the three strategies.

Representative nitrogen N1s core level regions of the utilized molecules: (a) PDA, (b) SBMA, and (c) DMAPAA, with their deconvolution. These spectra can be deconvoluted as follows: (a) one peak at (400.0 ± 0.2) eV, (b) one peak at (401.5 ± 0.2) eV, (c) two peaks, the main at (398.8 ± 0.2) eV and the secondary at (400.7 ± 0.2) eV.

Representative nitrogen N1s core level regions of SBMA coating (d) strategy 1, (e) strategy 2, and (f) strategy 3, with their deconvolution. These spectra can be deconvoluted as follows: (d) two peaks, the main at (399.7 ± 0.2) eV can be attributed to the presence of PDA, and the secondary at (402.5 ± 0.2) eV can be attributed to the presence of SBMA; (e) two peaks, the main at (399.7 ± 0.2) eV can be attributed to the presence of PDA, and the secondary at (402.5 ± 0.2) eV can be attributed to the presence of SBMA; (f) four peaks, the main at (399.9 ± 0.2) eV can be attributed to the presence of PDA, the peak at (398.8 ± 0.2) eV can be attributed to the presence of DMAPAA, the peak at (400.9 ± 0.2) eV can be attributed to both DMAPAA and SBMA, the peak at (402.3 ± 0.2) eV can be attributed to the presence of SBMA.

The binding energy of nitrogen of the SBMA in the coatings (panels d, e, f) shows a small shift compared to the binding energy of the SBMA molecules alone (panel b). This shift can result from the interaction between the SBMA and the other molecules composing the coating.

PDA = polydopamine, SBMA = [2-(methacryloyloxy)ethyl]dimethyl-(3-sulfopropyl)ammonium hydroxide, DMAPAA = N-[3-(dimethylamino)propyl]acrylamide].


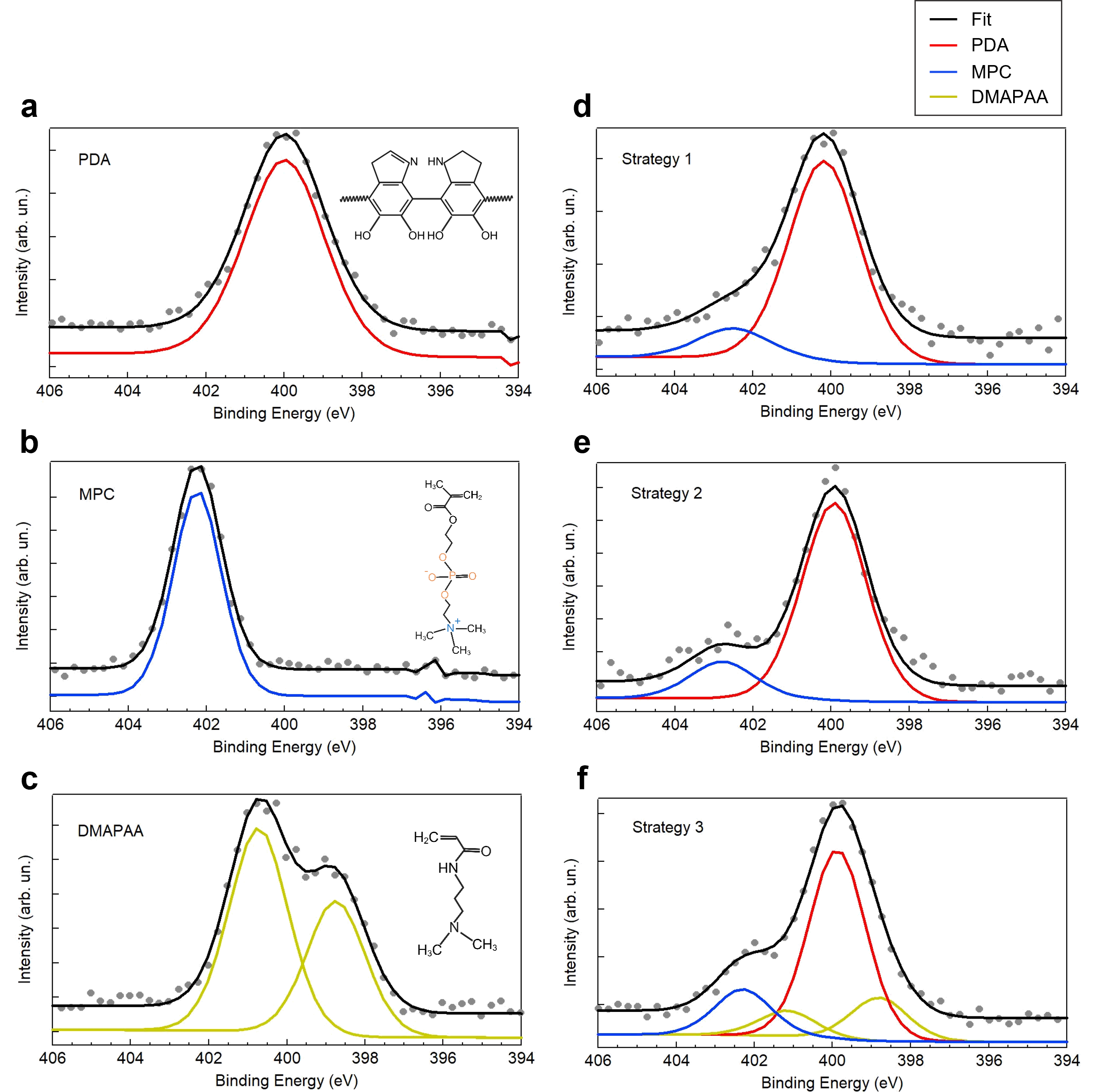


**Supplementary Figure S2.** XPS analysis of nitrogen signals confirms the presence of the MPC-based coatings obtained with the three strategies.

Representative nitrogen N1s core level regions of the utilized molecules: (a) PDA, (b) MPC, and (c) DMAPAA, with their deconvolution. These spectra can be deconvoluted as follows: (a) one peak at (400.0 ± 0.2) eV, (b) one peak at (402.2 ± 0.2) eV, (c) two peaks, the main at (398.8 ± 0.2) eV and the secondary at (400.7 ± 0.2) eV.

Representative nitrogen N1s core level regions of MPC coating (d) strategy 1, (e) strategy 2, and (f) strategy 3, with their deconvolution. These spectra can be deconvoluted as follows: (d) two peaks, the main at (400.2 ± 0.2) eV can be attributed to the presence of PDA, and the secondary at (402.5 ± 0.2) eV can be attributed to the presence of MPC; (e) two peaks, the main at (399.9 ± 0.2) eV can be attributed to the presence of PDA, and the secondary at (402.8 ± 0.2) eV can be attributed to the presence of MPC; (f) four peaks, the main at (399.9 ± 0.2) eV can be attributed to the presence of PDA, the peaks at (398.8 ± 0.2) eV and (401.2 ± 0.2) eV can be attributed both to the presence of DMAPAA, the peak at (402.3 ± 0.2) eV can be attributed to the presence of MPC.

PDA = polydopamine, MPC = 2-methacryloyloxyethyl phosphorylcholine, DMAPAA = N-[3-(dimethylamino)propyl]acrylamide].


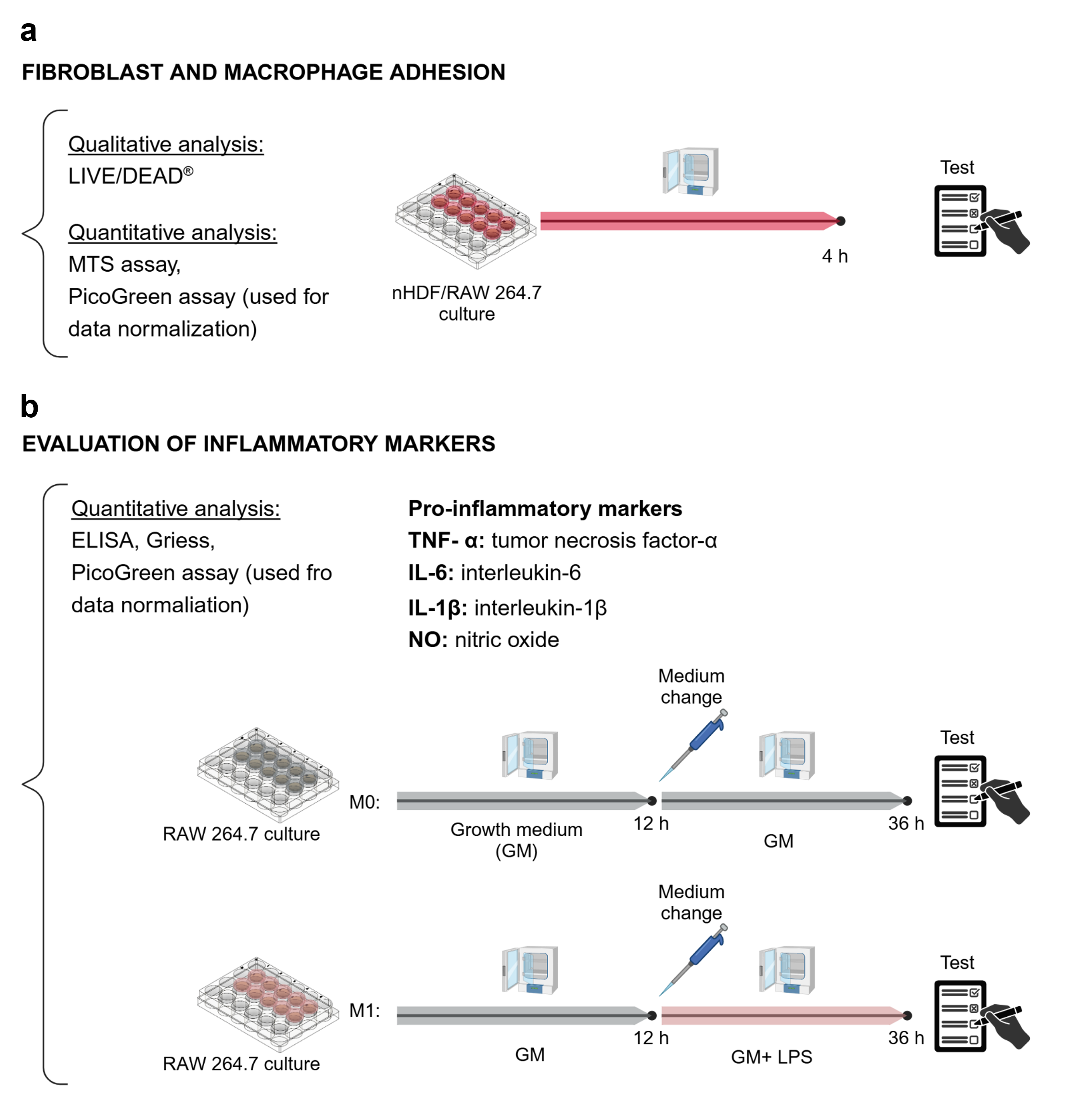


**Supplementary Figure S3**. Depiction of the in vitro tests and timeline of the experiments. (a) Cell adhesion. (b) Evaluation of pro-inflammatory markers. GM = growth medium, LPS = lipopolysaccharide, M0 = macrophage culture with M0 phenotype, M1 = macrophages skewed toward an M1-like phenotype (pro-inflammatory state) by adding LPS in the culture media


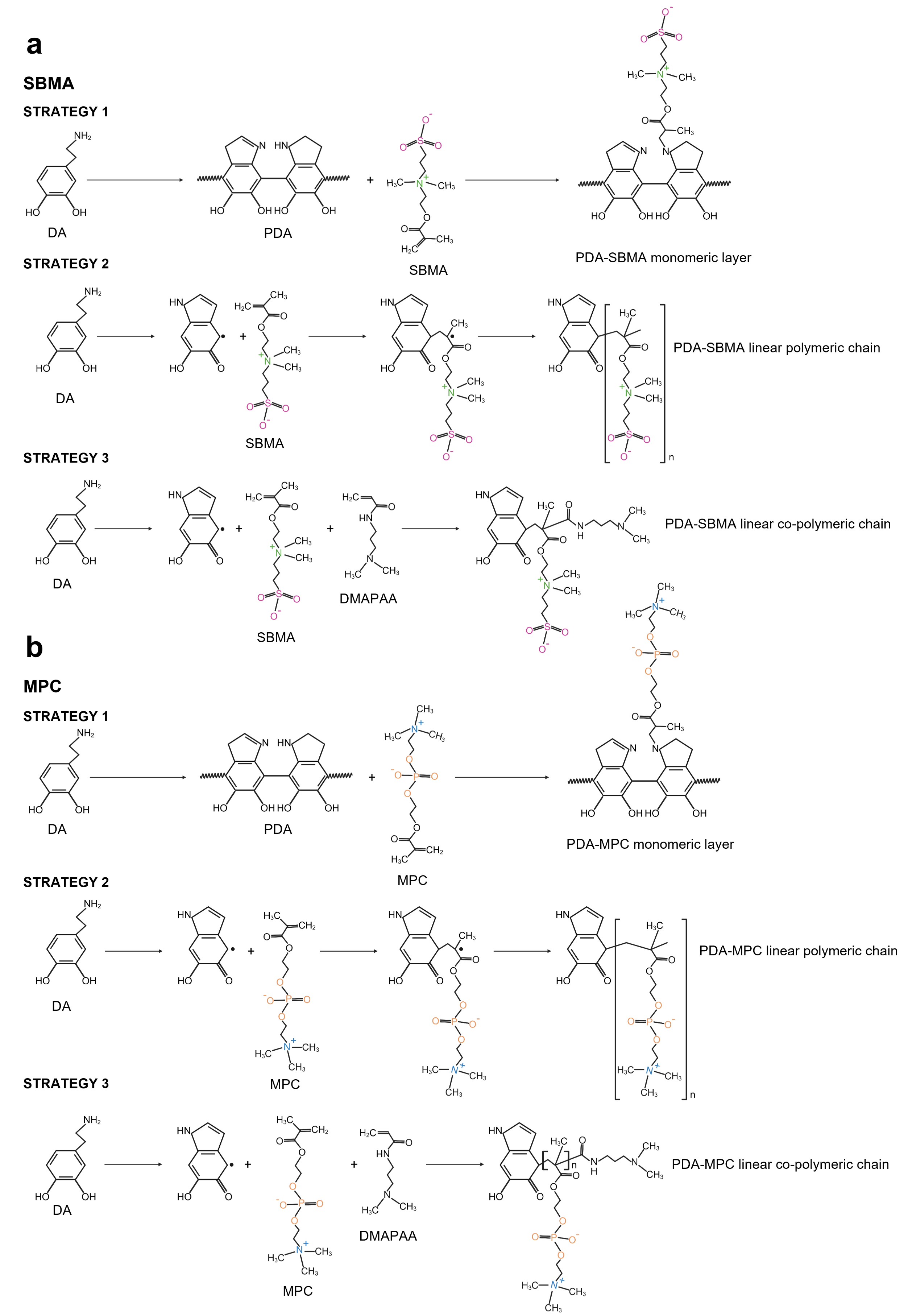


**Supplementary Figure S4.** Suggested chemical interaction mechanism between dopamine, zwitterions, and DMAPAA in coating fabrication, for the three coating strategies. (a) SBMA integration: in strategy 1, the acrylate moiety of SBMA undergoes Michael addition with the amine groups of PDA ^S1^. In strategy 2, the spontaneous auto-oxidation of dopamine generates radicals that trigger the free-radical polymerization of SBMA monomers ^S2^. In strategy 3, the spontaneous auto-oxidation of dopamine generates radicals that trigger the free-radical polymerization of SBMA and/or DMAPAA monomers. (b) MPC integration: in strategy 1, the acrylate moiety of MPC undergoes Michael addition with the amine groups of PDA ^S1^. In strategy 2, the spontaneous auto-oxidation of dopamine generates radicals that trigger the free-radical polymerization of MPC monomers ^S2^. In strategy 3, the spontaneous auto-oxidation of dopamine generates radicals that trigger the free-radical polymerization of MPC and/or DMAPAA monomers.

PDA = polydopamine, SBMA = [2-(methacryloyloxy)ethyl]dimethyl-(3-sulfopropyl)ammonium hydroxide, DMAPAA = N-[3-(dimethylamino)propyl]acrylamide], MPC = 2-methacryloyloxyethyl phosphorylcholine.

SUPPLEMENTARY REFERENCES

S1. Liu, C.-Y. & Huang, C.-J. Functionalization of Polydopamine via the Aza-Michael

Reaction for Antimicrobial Interfaces. Langmuir **32,** 5019–5028 (2016).

S2. Zhang, C. et al. Dopamine-Triggered One-Step Polymerization and Codeposition of

Acrylate Monomers for Functional Coatings. ACS Appl. Mater. Interfaces **9,** 34356–34366

(2017).
